# ADAM and ADAMTS proteases as integrative hubs in heart failure pathogenesis and therapy

**DOI:** 10.1016/j.isci.2026.116533

**Published:** 2026-06-26

**Authors:** Xinyu Wang, Yujie He, Yuyan Wang, Yue Liu, Xinyu Dai, Yunhao Xia, Lanzhi Du, Chang Li, Dan Zhang

**Affiliations:** 1 Department of Rehabilitation Medicine, Southwest Medical University, Rehabilitation Medicine and Engineering Key Laboratory of Luzhou, Luzhou 646000, Sichuan, China; 2 Electrophysiological Key Laboratory of Sichuan Province, Institute of Cardiovascular Research, Southwest Medical University, Luzhou 646000, Sichuan, China

**Keywords:** ADAM, ADAMTS, heart failure, pathogenesis, therapy

## Abstract

Heart failure (HF) arises from multifactorial disruptions in cellular signaling, inflammation, and tissue remodeling beyond classical neurohormonal regulation. Increasing evidence highlights ADAM and ADAMTS metalloproteinases as central integrators of inflammatory, fibrotic, and hypertrophic pathways. This review establishes a dual integrative framework. Horizontally, distinct protease family members converge within each pathological axis, including inflammation, fibrosis, hypertrophy and remodeling, metabolism, and angiogenesis, to drive disease progression. Vertically, individual proteases, such as ADAM17, coordinate multiple axes through various effectors that activate distinct downstream pathways, exhibiting both detrimental and protective functions. These mechanistic insights guide the development of selective inhibitors to circumvent pitfalls associated with broad-spectrum MMP targeting. Additionally, emerging ADAM and ADAMTS family members show promise as diagnostic and prognostic biomarkers in cardiovascular diseases (CVDs). By synthesizing mechanistic depth with translational relevance, this framework reveals protease-centered opportunities for precision intervention and outlines essential future research directions.

## Graphical abstract

**Figure:**
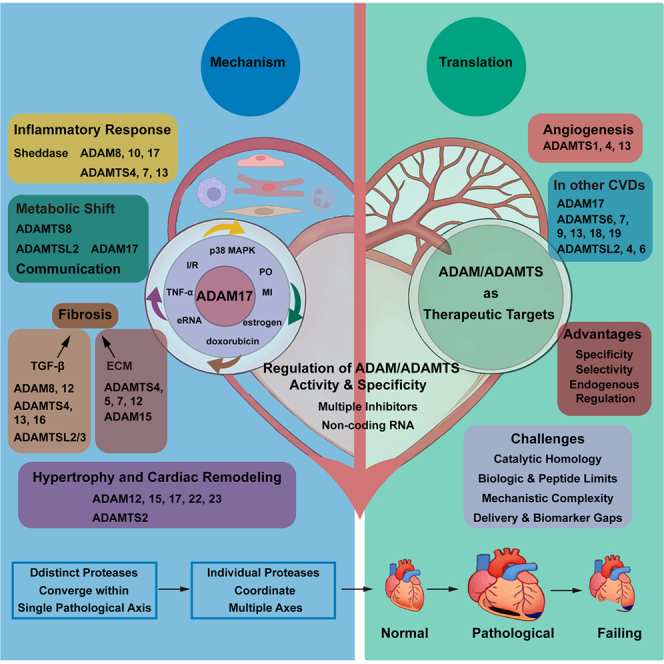

## Teaser

Cardiovascular medicine; Health sciences; Medicine

## Introduction

Heart failure (HF) is a severe manifestation in the middle-to-late stages of various cardiovascular diseases (CVDs), posing significant risks to life. Due to high morbidity, mortality, and poor prognosis, HF has emerged as a critical global public health concern. Epidemiological data indicate that the global prevalence of HF among adults is between 1% and 3%, with incidence increasing significantly with age. Despite ongoing advances in treatment aimed at improving HF outcomes, the mortality rate remains high, with five-year survival below 50% and five-year mortality reaching 75% in certain cohorts.^1^^,^^2^ Although improving survival rates is crucial, this alone cannot eliminate the disease burden, highlighting an urgent need for enhanced prevention and management. Current treatments only relieve symptoms, rather than reverse myocardial remodeling, underscoring the necessity for new therapies targeting pathological mechanisms.^3^

Chronic HF, the final stage of progressive cardiac dysfunction, involves a dynamic shift from compensatory adaptation to decompensation. In the early stages, the Frank-Starling mechanism increases preload, myocardial hypertrophy (MH) raises afterload, and activation of the renin-angiotensin-aldosterone system (RAAS) maintains hemodynamic stability through neurohumoral regulation. However, sustained compensation leads to a vicious cycle of myocardial remodeling, characterized by pathological MH and interstitial fibrosis, increasing ventricular stiffness, and ultimately exacerbating HF.^4^ This pathological transformation involves a cascade of multi-level regulatory networks. At the molecular level, energy metabolism, immune and inflammatory responses, tissue ischemia, extracellular matrix (ECM) remodeling, and small-molecule mediators collectively contribute to HF.^5^ Within the immune-inflammatory microenvironment, pro-inflammatory factors such as tumor necrosis factor-α (TNF-α) and interleukin-6 (IL-6) amplify tissue damage via the toll-like receptor 4 (TLR4) nuclear factor kappa-light-chain-enhancer of activated B cells (NF-κB) signaling pathway.^6^^,^^7^ Disruption of ECM homeostasis, driven by an imbalance between matrix metalloproteinases (MMPs) and their inhibitors, leads to abnormal collagen deposition and altered ventricular wall mechanics.^8^ Concurrently, neuroendocrine compensation fails as combined signaling from angiotensin II (Ang II) and endothelin-1 (ET-1) promotes vasoconstriction and fluid retention, generating a pathological positive feedback loop.^9^ These simultaneous processes are mediated centrally by proteolytic activity and ECM interactions.

MMPs have been extensively studied as potential biomarkers of CVD progression, especially in cardiac remodeling and HF following myocardial infarction (MI). MMPs degrade the ECM to produce matrix cryptins, which play essential roles in HF development post-MI and may serve as novel therapeutic targets. However, the broad substrate specificity of MMPs and their strict regulation by tissue metalloproteinase inhibitors (TIMPs) limit their therapeutic specificity.^8^^,^^10^^,^^11^ Conversely, the disintegrin and metalloproteinase (ADAM) family has dual functions. Its metalloproteinase domain mediates proteolytic activity, while its disintegrin domain facilitates integrin-mediated cell adhesion. This structural feature distinguishes ADAMs from MMPs, which primarily degrade ECM components. The ADAM with thrombospondin motifs (ADAMTS) subfamily is characterized by one or more thrombospondin type 1 repeats (TSR) within its complex C-terminal ancillary domains. Through these domains, ADAMTS proteases directly target ECM components such as proteoglycans. This structural complexity allows precise regulation of ECM remodeling under pathological conditions by controlling enzyme-substrate interactions and cellular localization. ADAMTS proteases also participate in pathological processes, including intercellular communication, signal transduction, inflammatory responses, and cell migration.^12^^,^^13^^,^^14^

Research suggests that ADAM family members construct a cardiovascular protection/damage axis through distinct functional roles. For instance, ADAM17 activates the epidermal growth factor receptor (EGFR) pathway to promote pathological MH,^15^^,^^16^ whereas ADAM10 maintains myocardial cell survival via Notch signaling.^17^^,^^18^ This functional diversity enables the ADAM/ADAMTS family to drive myocardial fibrosis and contractile dysfunction, alleviating mechanical stress through ECM remodeling and establishing a unique balance between HF progression and compensation. ADAMTS13 has a crucial regulatory role in various CVDs, including MI,^19^^,^^20^ ischemic stroke,^19^^,^^21^ HF,^22^ and pulmonary hypertension.^23^ ADAMTS13 preserves microcirculation by cleaving von Willebrand factor (VWF), and functional defects can worsen inflammatory damage following MI. Reduced ADAMTS13 activity directly correlates with increased MI risk and synergistically elevates MI incidence in women using oral contraceptives.^19^ The ADAMTSL subfamily, meanwhile, exerts bidirectional regulatory effects on ventricular remodeling by assembling fibrin microfibril scaffolds and coordinating transforming growth factor β (TGF-β) signaling and ECM mechanical stability.^24^

This review focuses on the essential roles of the ADAM and ADAMTS protein families in CVDs, particularly HF. It systematically explores the multifaceted functions of the ADAM/ADAMTS family in HF, from the initial signaling for remodeling via ECM degradation, through the mid-stage amplification of fibrosis driven by inflammatory cascades, to full-cycle regulation of compensatory matrix remodeling in late-stage disease. Furthermore, the review discusses ADAM/ADAMTS family members’ roles in angiogenesis and their potential as biomarkers, alongside the selectivity and translational opportunities and challenges associated with ADAM/ADAMTS inhibitors. By integrating existing evidence, this study provides a comprehensive perspective on HF pathogenesis, establishing important theoretical foundations and directions for developing targeted therapeutic strategies.

## Overview of the structural characteristics and biological functions of the ADAM and ADAMTS families

ADAM and ADAMTS are zinc-dependent metalloproteinases that cleave transmembrane proteins and ECM components, thereby regulating cell adhesion, signaling, and tissue remodeling.^14^^,^^25^ ADAMs primarily mediate the shedding of cell surface proteins, including growth factors, cytokines, and adhesion receptors, to modulate cell migration, proliferation, and differentiation. ADAMTS proteases mainly process ECM components such as proteoglycans and procollagen, thereby contributing to ECM organization, degradation, and remodeling.^13^^,^^26^ Dysregulation of ADAM and ADAMTS proteins disrupts the cellular microenvironment and is implicated in CVDs.^27^^,^^28^ To date, 37 ADAMs have been identified in the rat genome, 34 in the mouse genome, and 21 in the human genome, among which 13 possess proteolytic activity.^13^ The human ADAMTS family comprises 19 catalytically active members and 7 inactive homologs. In mice, the PAPLN gene is absent, whereas in rats, both the ADAMTS19 and PAPLN genes are missing.^29^

**Figure 1:**
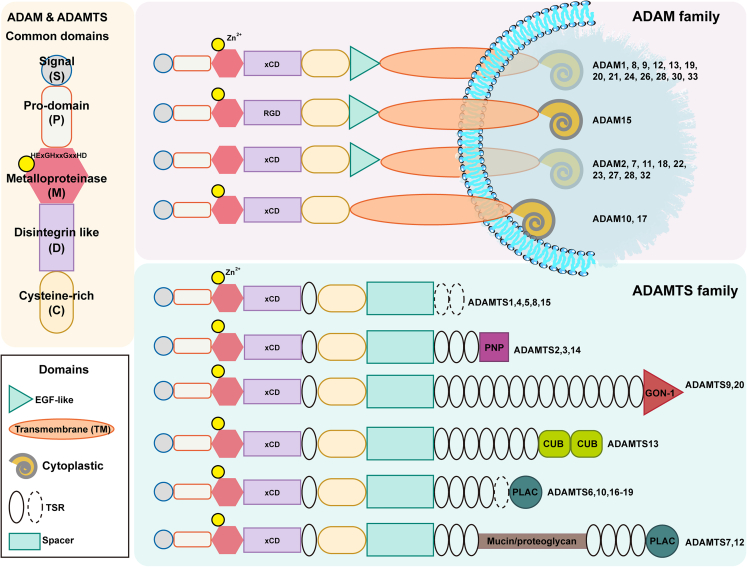
The structural schematics of ADAM and ADAMTS family proteases ADAMs and ADAMTS have five common domains: the signal peptide, pro-domain, metalloproteinase domain, disintegrin-like domain, and cysteine-rich domain. ADAM members have an additional EGF-like region, transmembrane domain, and cytoplasmic tail. Based on a typical metalloprotease (M)-domain with the characteristic catalytic Zn^2+^ binding signature (HExGHxxGxxHD), the disintegrin D-domain is based on xCD/RGD sequence, and the EGF-like region, ADAM members can be classified into four types. ADAMTS members are similar to ADAMs but lack the EGF-like region, transmembrane domain, and cytoplasmic tail. Instead, they have a spacer and TSR. ADAMTS members can be classified into six types based on the ancillary domain and TSR number. CUB, GON-1, PLAC: ancillary domain of the ADAMTS family, and the most significant variability between ADAMTS members. CUB: abbreviation of C1r/C1s, Uegf, and Bmp1; D: disintegrin; EGF: epidermal growth factor; M: metalloprotease; TSR: thrombospondin repeat; PNP: procollagen N propeptidase.

Members of the ADAM family are primarily membrane-bound proteases, characterized by a pro-domain that inhibits enzymatic activity, a metalloproteinase domain responsible for proteolysis, a disintegrin domain involved in cell adhesion and signal transduction, a cysteine-rich domain, and a transmembrane and cytoplasmic domain, which may be absent in some members.^30^ ADAM family members are classified based on structural features, including the sequence motif (xCD or RGD) within the disintegrin domain, the catalytic Zn^2+^-binding motif in the metalloproteinase domain, and EGF-like domains, and are further distinguished by tissue-specific expression or the presence of pseudogenes (Figure 1).^31^ The ADAMTS family members are primarily secreted enzymes with structures similar to those of the ADAM family but lack transmembrane domains. Their C-terminus contains one or more characteristic TSR, which contribute to substrate specificity and localization.^31^^,^^32^ The systematic classification of ADAMTS family members follows the principle that the C-terminal specialized domains determine functional categories, whereas the number of TSRs modulates activity within each subfamily. Accordingly, ADAMTS members are categorized into six groups.^33^^,^^34^ The detailed structures of these categories are shown in Figure 1.

## Imbalance between activation and regulation of the inflammatory response

### ADAM family: microenvironmental sensing and shear regulation of membrane-anchored proteases

The ADAM family regulates substrate cleavage dynamically by sensing the inflammatory microenvironment, mediating pro-inflammatory factor release, immune cell infiltration, and inflammatory injury cascades. Imbalances in this function accelerate the deterioration of the myocardial inflammatory microenvironment, becoming a key driver of HF progression.^35^^,^^36^^,^^37^ ADAM8 is upregulated in cardiac macrophages of mice with sepsis-induced cardiomyopathy. It cleaves MerTK to generate soluble Mer (sMer), which activates the cytokine-cytokine receptor interaction pathway, enhances inflammatory responses, and impairs efferocytosis, ultimately leading to cardiac dysfunction. Macrophage-specific ADAM8 knockout downregulates this inflammatory pathway and mitigates these adverse effects.^38^ In the context of MI, ADAM8 expression is similarly upregulated in cardiac macrophages. This increase promotes phosphorylation of annexin A2 (ANXA2) at Ser26, thereby activating the mTOR signaling pathway, which inhibits autophagy and reduces angiogenic factor release, ultimately promoting inflammation. Autophagy activation induces macrophage polarization toward a reparative phenotype, enhances vascular endothelial growth factor A (VEGFA) secretion to improve angiogenesis, and suppresses NLRP3 inflammasome activation to reduce IL-1β and IL-18 release. These changes collectively improve cardiac function. Conversely, ADAM8 overexpression inhibits ANXA2, sustains mTOR activity, blocks autophagy, and exacerbates inflammatory injury and cardiac remodeling.^39^

As a critical membrane-bound protease, ADAM17 dynamically regulates transmembrane protein cleavage through its microenvironment-sensing ability, exhibiting dual roles with spatiotemporal specificity in HF progression. A recent study demonstrated that extracellular RNA (eRNA) released from cardiomyocytes (CMs) during ischemia/reperfusion (I/R) injury triggers ADAM17-mediated TNF-α shedding, directly linking I/R-induced necrosis to protease-driven inflammation.^40^ Following acute MI, ADAM17 becomes activated via the β2-AR/Gαi-p38 MAPK/ERK pathway, releasing TNF-α and upregulating intercellular adhesion molecule 1 (ICAM-1), which promotes neutrophil infiltration and CM apoptosis. TNF-α knockout reduces inflammatory damage at this stage.^35^ ADAM17-mediated ACE2 cleavage decreases Ang 1–7, abolishing its Mas receptor-dependent suppression of NF-κB and pro-inflammatory cytokines (TNF-α, IL-6), thereby aggravating myocardial inflammation. Concurrently, accumulated Ang II activates Ang II type 1 receptor (AT1R)/NOX2 signaling to increase ROS production and amplify inflammatory cascades.^41^ ADAM17 also cleaves membrane-bound TNF-α to generate soluble TNF-α, exacerbating ventricular remodeling via the inflammation-apoptosis signaling axis.^42^ Additionally, ADAM17 serves as a key link between neuroinflammation and cardiac injury, exacerbating both central nervous system inflammation and cardiac damage by cleaving transmembrane TNF-α into bioactive soluble TNF-α and activating downstream NF-κB and ERK1/2 signaling pathways, thus further fueling this cycle.^36^^,^^37^ Estrogen regulates ADAM17 membrane localization and activity through the GPER-1/PI3K pathway, inhibiting stress-induced cardiac inflammation and injury.^43^ The synergistic effect of ADAM17 and MMP9 is particularly significant in the TIMP-3 deficiency model, where both exacerbate matrix degradation and inflammatory responses.^35^ Although ADAM17 is predominantly recognized for pro-inflammatory functions through cytokine shedding, such as TNF-α, it also plays a context-dependent protective role by regulating death receptor availability. ADAM17 mediates TNFR1 shedding in response to sphingomyelinase-induced phosphatidylserine externalization, protecting endothelial cells (ECs) from TNF-α-induced apoptosis.^44^

ADAM10 exhibits context-dependent functions in cardiovascular pathophysiology, demonstrating protective and harmful effects across various disease states. In ECs, ADAM10 provides protective outcomes through several mechanisms. It regulates coronary endothelial differentiation and maturation via Notch and EGFR signaling; its deficiency results in coronary defects, cardiac dysfunction, elevated venous markers, and disrupted EGFR signaling.^17^ Additionally, ADAM10 protects against hypoxia/reoxygenation (H/R) injury by activating Notch signaling, thereby reducing oxidative stress and apoptosis.^18^ It also cleaves LOX-1, decreasing membrane retention and endothelial sensitivity to oxidized LDL (oxLDL), suppressing downstream PI3K-AKT and MAPK inflammatory pathways, reducing the release of adhesion molecules and chemokines, and attenuating inflammatory infiltration.^45^ Conversely, during myocardial ischemia, elevated ADAM10 levels have detrimental effects through the shedding of C-X3-C motif chemokine ligand 1 (CX3CL1). Pharmacological inhibition or genetic deletion of ADAM10 improves survival, cardiac function, and reduces scarring in mouse models by preventing CX3CL1 cleavage. This action subsequently reduces IL-1β-mediated inflammation and neutrophil mobilization from the bone marrow. Targeted inhibition of ADAM10 effectively disrupts the ADAM10-CX3CL1-neutrophil axis, reduces excessive inflammation, preserves myocardial function, and has long-term benefits through short-term treatment.^46^ Therapeutic intervention targeting this axis consistently improves post-MI outcomes by mitigating inflammation and neutrophil infiltration.

**Figure 2:**
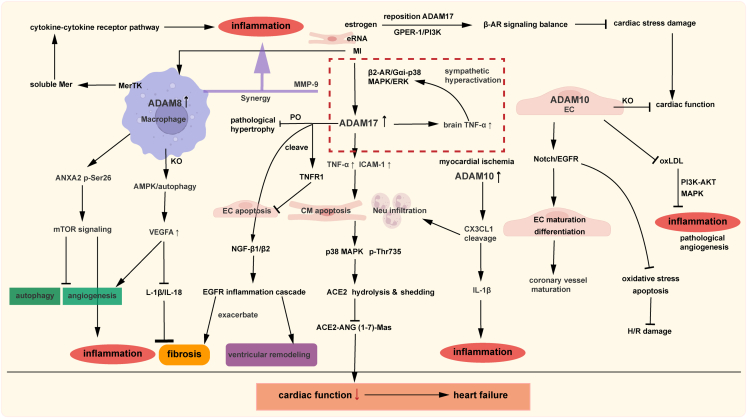
ADAMs-mediated inflammatory crosstalk in heart failure. ADAM8 in macrophages cleaves MerTK to release sMer, which amplifies inflammatory signaling It suppresses autophagy and angiogenesis via ANXA2-Ser26/mTOR signaling. Genetic ablation activates AMPK/autophagy and upregulates VEGFA, attenuating fibrosis while promoting angiogenesis. ADAM17 as a central driver of HF through: (1) post-infarction upregulation of TNF-α/ICAM-1, inducing CM apoptosis and neutrophil infiltration. (2) p38/AMPK-Thr735 phosphorylation-mediated ACE2 shedding, inhibiting ACE2-Ang(1–7)-Mas cardioprotection. (3) β-NGF cleavage-dependent activation of EGFR, inflammation, and profibrotic cascades. (4) Brain-derived TNF-α triggers sympathetic overactivation via β2-AR/Gαi-p38 MAPK. Estrogen induces GPER-1/PI3K-dependent perinuclear translocation, modulating β-AR signaling. ADAM10 exhibits dual roles. ADAM10 promotes EC maturation and attenuates H/R injury via Notch signaling, while suppressing oxLDL-induced angiogenesis. In MI, ADAM10 cleavage of CX3CL1 exacerbates inflammation by enhancing IL-1β and neutrophil recruitment. ACE2: angiotensin-converting enzyme 2; AKT: AK strain transforming/protein kinase B; AMPK: adenosine monophosphate-activated protein kinase; ANXA2: annexin A2; CM: cardiomyocyte; CX3CL1: C-X3-C motif chemokine ligand 1; EC: endothelial cell; EGFR: epidermal growth factor receptor; GPER-1: G protein-estrogen receptor-1; ERK: extracellular signal-regulated kinase; ICAM-1: intracellular adhesion molecule-1; MI: myocardial infarction; IL-1β: interleukin-1β; Neu: neutrophil; oxLDL: oxidized low-density lipoprotein; MI: myocardial infarction; PI3K: phosphatidylinositol 3-kinase; TNF-α: tumor necrosis factor-α; VEGFA: vascular endothelial growth factor; β-AR: β-adrenergic receptor; NGF-β: β-nerve growth factor.

In the acute phase of ischemia (early MI), ADAM10 expression rapidly increases in CMs, initiating IL-1β-dependent neutrophil recruitment by cleaving CX3CL1, thus exacerbating inflammation and myocardial damage. In contrast, during chronic remodeling or homeostasis, endothelial ADAM10 cleaves the oxLDL receptor LOX-1 to suppress endothelial inflammation, while concurrently activating Notch signaling to promote coronary arterial maturation and inhibit pathological neovascularization. Through these mechanisms, endothelial ADAM10 provides protective effects against atherosclerosis (AS) and maintains cardiac structural integrity. These observations clarify previous inconsistencies by identifying ADAM10 as an adaptive cardiac regulator whose function shifts based on context-specific factors such as pathological stage, cell type, and substrate specificity, rather than intrinsic functional contradictions. The ADAM family is a crucial mediator of microenvironmental sensing and shear-induced regulation of membrane-bound proteases. Details on the role of ADAMs in inflammatory signaling in HF are presented in Figure 2.

### ADAMTS-Mediated Inflammatory Cascades in HF

**Figure 3:**
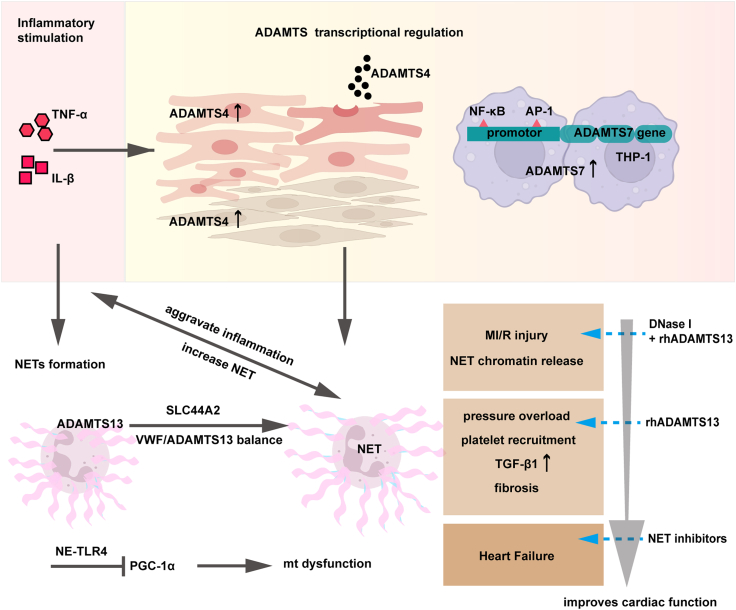
ADAMTS-mediated inflammatory cascades in HF. TNF-α/IL-1β synergistically induce versican/ADAMTS4 mRNA and protein secretion in CM/FB Pro-inflammatory NF-κB/AP-1 bind ADAMTS7 promoter; TNF-α drives monocyte/macrophage expression. ADAMTS13-SLC44A2-NETs axis impairs cardiac function via NET-TLR4-PGC-1α mitochondrial dysfunction. rhADAMTS13 reduces TGF-β1-mediated fibrosis via suppressing VWF-dependent platelet recruitment. AP-1: activator protein-1; CM: cardiomyocyte; FB: fibroblast; NETs: neutrophil extracellular traps; NF-κB: nuclear factor kappa-B; HF: heart failure; PGC-1α: peroxisome proliferator-activated receptor gamma coactivator 1-α; TLR4: toll-like receptor 4; VWF: von Willebrand factor.

Similarly, the role of ADAMTS family members is illustrated in Figure 3. Studies have shown that the inflammatory TNF-α and IL-1β synergistically increase versican and ADAMTS4 mRNA expression in neonatal cardiomyocytes (NCMs) and cardiac fibroblasts (CFBs), and enhance ADAMTS4 secretion by NCMs.^47^ Furthermore, the ADAMTS7 promoter contains binding sites for pro-inflammatory transcription factors, including NF-κB and activator protein-1 (AP-1). TNF-α induces the expression of ADAMTS7 in the human monocyte/macrophage cell line THP-1. Chromatin immunoprecipitation analysis has confirmed the binding of NF-κB and AP-1 to their respective sites within the ADAMTS7 promoter, indicating that ADAMTS7 is transcriptionally regulated by inflammatory stimuli under pathological conditions.^48^

Beyond transcriptional regulation, neutrophil extracellular traps (NETs) critically contribute to HF pathogenesis via the VWF-SLC44A2-NET axis. NETs suppress PGC-1α via the neutrophil elastase (NE)-TLR4 pathway, leading to mitochondrial dysfunction in CMs. Importantly, VWF/ADAMTS13 modulates NET formation through SLC44A2. Targeting this axis or using NET inhibitors has been shown to improve cardiac function, highlighting potential therapeutic strategies for HF.^22^ In myocardial I/R injury, chromatin release from NETs further aggravates tissue damage. The combined administration of DNase I and recombinant human ADAMTS13 (rhADAMTS13) significantly enhances cardiac function by promoting chromatin clearance and inhibiting vWF-mediated leukocyte recruitment.^49^

## The ADAM/ADAMTS family modulates cardiac fibrosis via TGF-β signaling and ECM dynamics

**Figure 4:**
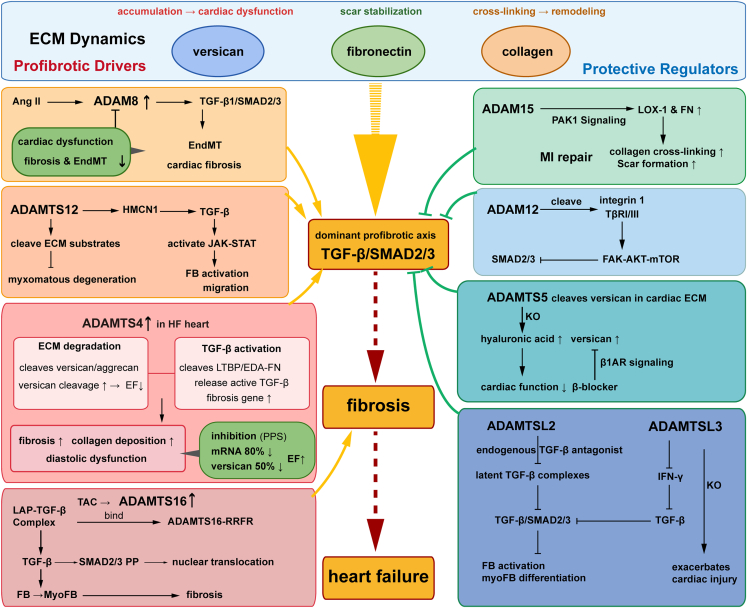
ADAM/ADAMTS family exerts either pro-fibrotic or anti-fibrotic effects by modulating ECM homeostasis and the TGF-β signaling axis The profibrotic drivers such as ADAM8, ADAMTS4, 12, and 16, which are elevated in different pathological processes, always activate the dominant profibrotic axis TGF-β/SMAD2/3 signaling and its related molecules, leading to fibrosis and worsening HF. Inhibiting them attenuates the pathological process and improves heart function. ADAM12, 15, ADAMTS5, L2, and L3 have a cardioprotective role through regulating ECM molecules and inhibiting TGF-β signaling. Ang II: angiotensin 2; ECM: extracellular matrix; EndMT: endothelial-mesenchymal transition; FAK: focal adhesion kinase; FB: fibroblast; FN: fibronectin; KO: knock out; LOX1: lectin-like oxidized low-density lipoprotein receptor-1; LTBP: latent TGF-β protein; PAK1: p21 activated kinase 1; TAC: transverse aortic constriction; TGF-β: transforming growth factor β; TβRI/III: TGF-β receptor I/III.

Cardiac fibrosis, characterized by excessive ECM deposition, significantly impairs cardiac function and represents a critical aspect of HF-associated pathological remodeling. The TGF-β signaling pathway, involving its ligands, receptors, and downstream SMAD-dependent and non-canonical pathways, centrally regulates cardiovascular homeostasis, injury repair, and disease progression.^50^ ADAM and ADAMTS proteins influence fibrosis through two interconnected mechanisms: modulation of TGF-β signaling and direct ECM remodeling (Figure 4).

ADAM8 promotes Ang II-induced cardiac fibrosis and endothelial-to-mesenchymal transition (EndMT) by activating TGF-β1/SMAD2/3 signaling.^51^ Elevated ADAMTS4 expression and activity occur in human failing hearts. ADAMTS4 directly cleaves ECM proteins such as extra domain A-fibronectin (EDA-FN) and latent TGF-β-binding protein 1 (LTBP1), releasing latent TGF-β and subsequently activating the TGF-β signaling cascade. This action promotes collagen deposition by CFBs, causing fibrosis and cardiac dysfunction. Pharmacological inhibition of ADAMTS4 significantly reduces myocardial collagen content, improves cardiac function, and decreases mortality in rat models of pressure overload (PO)-induced injury, highlighting its potential as a therapeutic target, especially for fibrosis-driven HF subtypes.^52^ ADAMTS16 directly binds to latency-associated peptide (LAP)-TGF-β via its RRFR motif, stimulating TGF-β/SMAD2/3 signaling in PO models, thereby inducing myofibroblast (myoFB) differentiation and collagen accumulation. Mimic peptides of the RRFR motif exacerbate fibrosis, whereas TGF-β neutralizing antibodies counteract this effect.^53^ Recombinant human ADAMTS13 (rhADAMTS13) attenuates PO-induced remodeling by reducing VWF-mediated platelet recruitment, a key source of profibrotic TGF-β1.^54^ Conversely, certain family members act as endogenous inhibitors of TGF-β signaling. ADAMTSL2, prominently secreted by CFBs and significantly upregulated in failing hearts, negatively regulates TGF-β by reducing latent TGF-β complex production and activation, rather than directly inhibiting active TGF-β. ADAMTSL2 strongly inhibits myoFB differentiation and CFB profibrotic phenotypes such as proliferation, migration, and contraction.^55^ Similarly, ADAMTSL3 negatively modulates TGF-β signaling, inhibiting abnormal ECM remodeling and attenuating cardiac fibrosis and dysfunction following PO. IFN-γ induces ADAMTSL3 expression, forming an IFN-γ/ADAMTSL3/TGF-β inhibitory axis that confers anti-inflammatory and anti-fibrotic effects. Loss of ADAMTSL3 exacerbates cardiac injury and mortality.^56^ Additionally, ADAM12 mitigates PO-induced hypertrophy and fibrosis by cleaving integrin β1 and TGF-β receptors type I and III, inhibiting excessive activation of downstream pathways including FAK-AKT-mTOR, ERK, and SMAD2/3.^57^

Direct ECM remodeling by these proteases also significantly impacts fibrosis. ADAMTS4 promotes fibrosis through ECM degradation, and its inhibition restores cardiac function.^52^ ADAMTS4 deficiency leads to versican accumulation and dysfunction, while versicanase inhibition by pentosan polysulfate (PPS) reduces fibrosis and improves cardiac contractility.^47^ ADAMTS12 promotes fibrosis by cleaving hemicentin-1 (HMCN1), disrupting its anchoring function, and cooperating with TGF-β signaling to activate JAK-STAT pathways, thereby driving FB activation and migration.^58^ ADAMTS5 is essential for versican cleavage in cardiac tissue. Its catalytic deficiency causes versican and hyaluronic acid accumulation, impaired cardiac function, and reduced expression of intercellular communication proteins. β-blockers, through the inhibition of β1AR signaling, decrease versican deposition, identifying the ADAMTS5-versican axis as a therapeutic target in ischemic HF.^59^

However, context-dependent protective roles are evident in cardiac valves, where ADAMTS12 and ADAMTS7 prevent myxomatous degeneration through ECM substrate cleavage and inhibition of excessive TGF-β activation.^60^ ADAM15 stabilizes collagen fibers post-MI via the PAK1/LOX-1/FN axis, protecting against rupture, while its deficiency results in disordered collagen structure.^61^

ADAM and ADAMTS proteins exhibit dual, context-dependent roles in cardiac fibrosis. Selective inhibition (e.g., ADAMTS4,^47^^,^^52^ ADAM8^51^) or enhancement of protective factors (e.g., ADAMTSL2/3,^55^^,^^56^ ADAM15^61^) holds potential for mitigating fibrotic remodeling in HF. Future studies should clarify the cell-specific actions and temporal dynamics of these proteases within the TGF-β/ECM axis to enable precise therapeutic interventions for CVDs.

## ADAM/ADAMTS-mediated metabolic dysregulation and communication in HF

**Figure 5:**
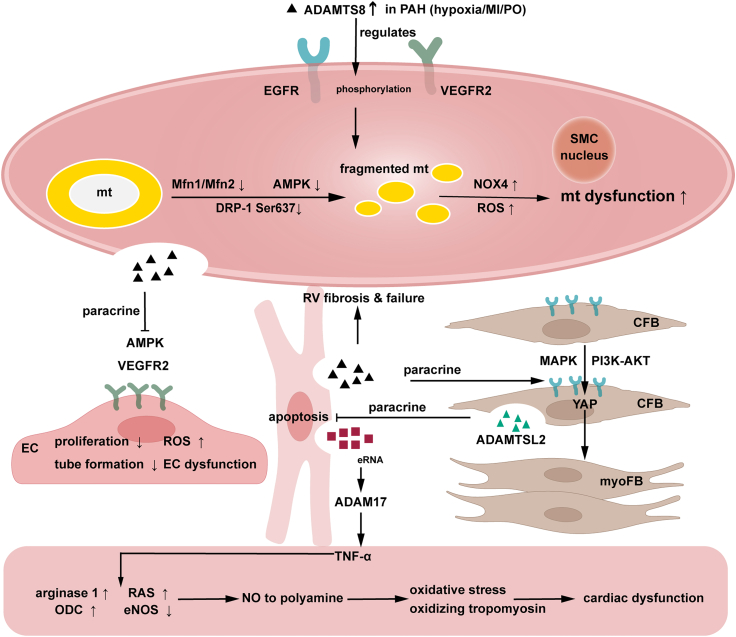
ADAM/ADAMTS-mediated metabolic dysregulation and communication in HF ADAMTS8 upregulation impairs mitochondrial fusion (*Mfn1*/*2* downregulation) and induces fragmentation. Paracrine ADAMTS8 from PASMCs impairs EC function via VEGFR2 and AMPK suppression. CM-derived ADAMTS8 directly enhances RV fibrosis and failure, while activating CFB through PI3K-AKT/MAPK-YAP signaling and CM-FB paracrine crosstalk, driving fibrosis. eRNA activates ADAM17 to release TNF-α, which shifts arginine metabolism from NO to polyamines, causing oxidative stress and contractile dysfunction. However, CFB-derived ADAMTSL2 inhibits CM apoptosis through paracrine signaling. DRP-1: dynamin-related protein 1; CFB: cardiac fibroblasts; eRNA: extracellular RNA; mt: mitochondria; PASMC: pulmonary arterial smooth muscle cell; ODC: ornithine decarboxylase; PO: pressure overload; RAS: renin-angiotensin system; ROS: reactive oxygen species; RV: right ventricular; VEGFR2: vascular endothelial growth factor receptor 2; YAP: yes-associated protein.

The role of ADAMTS8 in metabolic dysfunction and cellular communication positions it as a pivotal regulator of mitochondrial function in HF, as depicted in Figure 5. Elevated levels of ADAMTS8 occur in patients with pulmonary arterial hypertension (PAH) and in murine hypoxia-induced PAH models. Omura et al. identified ADAMTS8 as a pathogenic factor in PAH and right ventricular (RV) failure. Notably, ADAMTS8 is significantly elevated in pulmonary artery smooth muscle cells (PASMCs) from patients with PAH and RV tissues in rodent models. ADAMTS8 promotes PASMC proliferation and migration by inhibiting AMPK signaling and triggering mitochondrial fragmentation. Additionally, PASMC-derived ADAMTS8 suppresses VEGFR2 and AMPK in ECs via paracrine signaling, causing endothelial dysfunction, whereas CM-derived ADAMTS8 directly enhances RV fibrosis and failure. Treatment with mebendazole lowers ADAMTS8 expression, alleviating PAH and RV failure in animal models, highlighting its therapeutic potential.^62^

Extending these observations, Zha et al. reported that ADAMTS8 expression is elevated in myocardial tissue from patients with dilated cardiomyopathy (DCM) and animal models after MI or transverse aortic constriction (TAC). Increased ADAMTS8 correlates with elevated fibrosis markers, including α-SMA and collagen I. As a CM-secreted paracrine mediator, ADAMTS8 induces mitochondrial dysfunction in CFBs, reducing DRP-1 Ser637 phosphorylation and downregulating mitochondrial fusion genes *Mfn1* and *Mfn2*. This process results in mitochondrial fragmentation and increased NOX4-mediated reactive oxygen species (ROSs) production, activating EGFR-dependent MAPK and PI3K-AKT pathways. Ultimately, these changes promote YAP activation, stimulating FB proliferation, migration, and differentiation into myoFBs. Consistent with earlier findings, mebendazole treatment reduces ADAMTS8 levels, ameliorates cardiac fibrosis, and improves left ventricular function, underscoring ADAMTS8’s role in fibrosis via CM-to-FB paracrine interactions.^63^ Although the direct receptor for ADAMTS8 remains unknown, both studies demonstrate its involvement in modulating growth factor receptor pathways, specifically, suppressing VEGFR2 in ECs and activating EGFR in CFBs. In PASMCs, ADAMTS8-driven AMPK inhibition, which in turn alters DRP-1 phosphorylation and mitochondrial fusion gene (*Mfn1*/*2*) expression, promotes mitochondrial dysfunction.^62^ In CFBs, ADAMTS8-activated EGFR signaling stimulates MAPK/AKT-dependent YAP activation and myoFB differentiation.^63^ Therefore, ADAMTS8 integrates extracellular proteolysis and intracellular metabolic signaling, positioning it as a conserved therapeutic target for CVDs characterized by mitochondrial dysfunction and tissue remodeling.^62^^,^^63^

Beyond ADAMTS8, ADAM17 also significantly regulates cardiac metabolic dysregulation. The ADAM17-TNF-α-renin-angiotensin system (RAS) axis crucially modulates early myocardial dysfunction following I/R by initiating arginine metabolism reprogramming. Damaged CMs release eRNA during I/R, activating ADAM17, which cleaves and releases membrane-bound TNF-α. Released TNF-α induces metabolic shifts via two mechanisms: directly increasing arginase 1 and ornithine decarboxylase (ODC) levels, and activating local RAS, which suppresses endothelial nitric oxide synthase (eNOS). Consequently, cardiac arginine metabolism shifts from nitric oxide (NO) production to polyamine synthesis, raising oxidative stress and oxidizing tropomyosin, directly impairing contractile function shortly after I/R. Notably, ADAM17 inhibition blocks TNF-α release, reverses transcriptional disruptions in arginine metabolism, and interrupts the pathogenic cascade. Conversely, early metabolic interventions temporarily improve function but fail to prevent long-term HF progression.^64^ Conversely, ADAMTSL2 functions as a cardioprotective factor through cellular communication. Significantly elevated after MI, CFB-secreted ADAMTSL2 prevents CM apoptosis via paracrine signaling. Mechanistically, ADAMTSL2 interacts with LRP6, promoting its phosphorylation and stabilizing β-catenin, facilitating its nuclear translocation. Overexpression of ADAMTSL2 reduces CM apoptosis and enhances cardiac function, demonstrating its therapeutic promise.^65^

## ADAM and ADAMTS-mediated hypertrophy and myocardial remodeling drive HF progression

**Figure 6:**
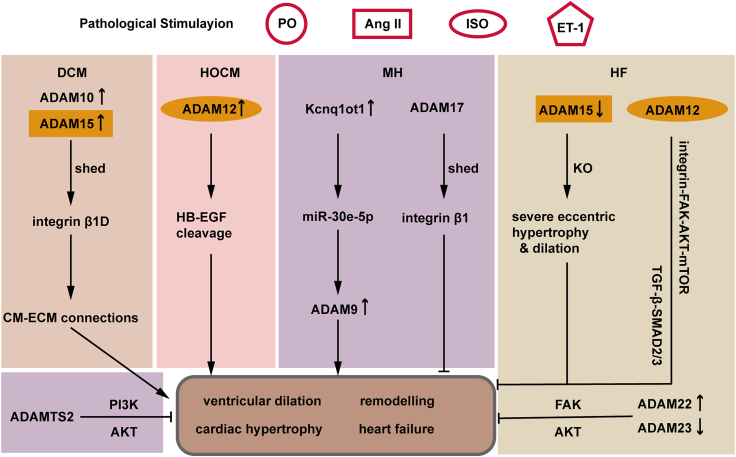
ADAM/ADAMTS Drive HF via Hypertrophy and Remodeling. ADAM15, 17, 22, 23, and ADAMTS2 protect against MH and adverse remodeling ADAM9 promotes hypertrophy via the Kcnq1ot1/miR-30e-5p axis. ADAM12 demonstrates dual roles: mediating MH through HB-EGF cleavage while attenuating HF via integrin-FAK-AKT-mTOR and TGF-β-SMAD2/3 suppression. ET-1: endothelin-1; HB-EGF: heparin-binding EGF; MH: myocardial hypertrophy; PI3K: phosphatidylinositol 3-kinase; ISO: isoproterenol.

Cardiac hypertrophy, characterized by pathological thickening of the ventricular wall, promotes HF by impairing ventricular function and triggering maladaptive remodeling. ADAM/ADAMTS proteases regulate cardiac hypertrophy and remodeling through multiple mechanisms, as illustrated in Figure 6. ADAM10 and ADAM15 are upregulated in DCM and may promote ventricular dilation by disrupting CM-ECM connections via integrin β1D shedding.^66^ However, ADAM15 is downregulated in end-stage human HF. ADAM15 deficiency in mice exacerbates eccentric hypertrophy and dilation following PO, suggesting a protective role.^67^ ADAM12 exhibits context-dependent effects in cardiac hypertrophy. It is specifically elevated in hypertrophic obstructive cardiomyopathy (HOCM) and functions as a pro-hypertrophic regulator.^66^ ADAM12 also mediates GPCR agonist-induced MH by cleaving heparin-binding EGF (HB-EGF), a process inhibited by the ADAM12-specific compound KB-R778.^68^ However, in PO-induced HF models, ADAM12 attenuates excessive hypertrophy and fibrosis, exerting protective effects by suppressing integrin-FAK-AKT-mTOR and TGF-β-SMAD2/3 signaling pathways. ADAM12 deficiency worsens HF progression.^57^ Thus, ADAM12 and ADAM15 have context-dependent roles in myocardial remodeling, with effects varying according to disease stage, pathological stimulus, and cellular environment. Under PO, ADAM17 limits pathological MH through integrin β1 cleavage. CM-specific ADAM17 knockdown exacerbates mechanical stress-induced hypertrophic responses and accelerates HF transition, confirming its protective role in PO cardiomyopathy.^69^ ADAM22 and ADAM23 show divergent expression patterns but converge on AKT pathway inhibition. ADAM22, upregulated in human failing hearts, negatively regulates MH. Cardiac-specific ADAM22 knockout exacerbates TAC-induced hypertrophy, while overexpression ameliorates it.^70^ In contrast, ADAM23 is downregulated in HF. ADAM23 deficiency aggravates PO-induced MH and fibrosis, whereas supplementation attenuates these phenotypes through FAK-AKT suppression.^71^ ADAMTS2 further extends this regulatory network, negatively controlling hypertrophy via PI3K/AKT inhibition. Its knockout exacerbates MH, while cardiac-specific overexpression alleviates pathological remodeling.^72^ Systems biology analysis identifies ADAMTS2 as a hub regulator of hypertrophy-related gene modules; silencing ADAMTS2 suppresses isoproterenol-induced CM hypertrophy.^73^ Long non-coding RNA Kcnq1ot1 is highly expressed in Ang II-induced MH. It competitively sequesters microRNA miR-30e-5p, relieving miR-30e-5p-mediated post-transcriptional repression of ADAM9. Elevated ADAM9 promotes MH, but the specific molecular mechanisms remain unclear, warranting further study.^74^

## Integrative protease hubs in HF: from single-pathway to multi-axis pathologies

### ADAM17 as a paradigm of multi-axis integration

**Figure 7:**
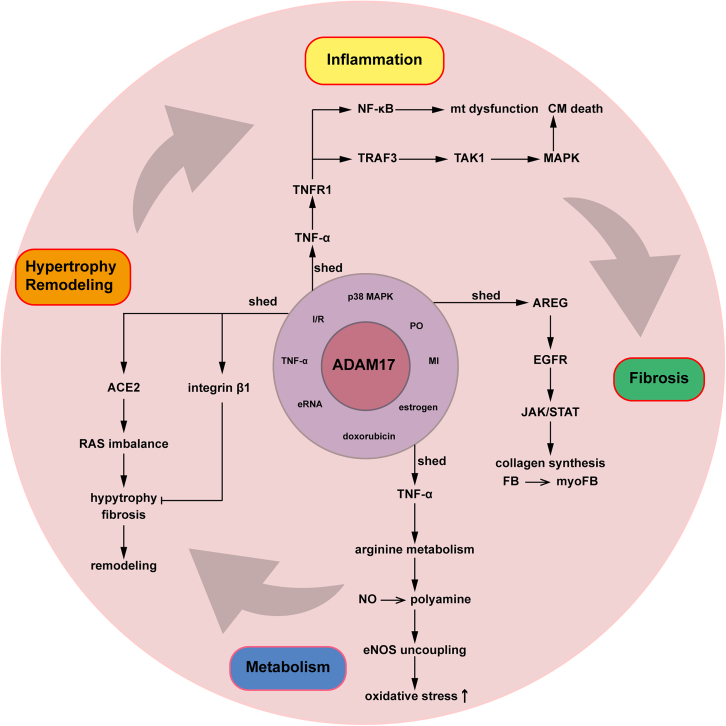
ADAM17 serves as a central hub linking multiple pathological processes in HF ADAM17 drives inflammation by shedding TNF-α, which upregulates TRAF3 and activates TAK1-MAPK signaling in CMs. It induces fibrosis by shedding AREG to activate EGFR-JAK/STAT signaling in FBs. It contributes to hypertrophy and remodeling through p38 MAPK-mediated phosphorylation and ACE2 shedding, leading to RAS imbalance. It also reprograms arginine metabolism from NO production toward polyamine synthesis via TNF-α, causing eNOS uncoupling and oxidative stress. ACE2: angiotensin-converting enzyme 2; AREG: amphiregulin; TAK1: TGF-β-activated Kinase 1; TRAF3: TNF receptor associated factor 3.

ADAM17 integrates multiple pathological processes in HF. Shedding of membrane-bound TNF-α acts as a common initiating event, activating diverse downstream pathways that link inflammation, fibrosis, hypertrophy, and metabolism (Figure 7).

ADAM17 functions as a unified upstream trigger and common effector. During myocardial I/R injury, damaged CMs release eRNA, directly activating ADAM17. Activated ADAM17 sheds TNF-α, creating a feedforward loop wherein eRNA and TNF-α reciprocally amplify tissue damage. Subsequently, TNF-α engages TNFR1, activating NF-κB signaling, inducing mitochondrial permeability transition pore opening, and promoting CM death.^40^ ADAM17 influences multiple axes of HF pathogenesis via TNF-α. In inflammation, doxorubicin activates ADAM17 in CMs through CCAAT/enhancer-binding protein beta (C/EBPβ), cleaving and activating TNF-α. Activated TNF-α upregulates TRAF3 through TNFR1, subsequently binding and activating TGF-β-activated kinase 1 (TAK1), initiating MAPK pathway activation, ultimately inducing CM apoptosis, cardiac remodeling, and HF.^42^ In metabolic remodeling, the ADAM17-TNF-α axis shifts arginine metabolism from NO production toward polyamine synthesis, leading to eNOS uncoupling and increased oxidative stress. This metabolic shift is reversible by the ADAM17 inhibitor TAPI.^64^ In FBs, ADAM17 sheds amphiregulin (AREG), activating EGFR on CFBs, stimulating JAK/STAT signaling, FB-to-myoFB differentiation, and collagen synthesis.^35^ Pathological stimuli activate p38 MAPK, phosphorylating ADAM17 at Thr735. Activated ADAM17 mediates membrane-bound ACE2 shedding, causing RAS imbalance, cardiac hypertrophy, fibrosis, remodeling, and eventually HF.^15^ Beyond the myocardium, ADAM17 in the paraventricular nucleus and sub-fornical organ promotes TNF-α release, triggering neuroinflammation and sustained sympathetic overactivity that exacerbates peripheral cardiac dysfunction.^36^^,^^37^

ADAM17 exhibits context-dependent roles in multiple pathological processes. It is not solely a driver of pathology but also exerts essential protective functions under specific conditions. Several factors determine this duality. First, the type of stress shapes ADAM17 function. In inflammation-driven pathologies, pro-inflammatory substrates predominate; under mechanical stress, integrin β1 becomes the primary target. During PO, ADAM17-mediated integrin β1 cleavage provides negative feedback to limit excessive hypertrophic signal amplification.^69^ Second, the cellular source matters. CM-derived ADAM17 supports post-ischemic angiogenesis by upregulating VEGFR2 expression via the TNF-α-NF-κB pathway, while neuronal ADAM17 drives sympathetic excitation.^36^^,^^75^ Third, the pathological stage influences the overall effect. Acute injury favors pro-inflammatory actions, while the repair phase engages pro-angiogenic functions.^15^^,^^75^ Multiple regulatory inputs fine-tune ADAM17 activity. p38 MAPK and ERK phosphorylate ADAM17 at Thr735, promoting activation.^15^^,^^36^ eRNA initiates the ADAM17-TNF-α axis, whereas RNase1-mediated eRNA degradation blocks this pathway.^40^ Estrogen downregulates membrane-bound ADAM17 via the GPER-1-PI3K pathway, reducing stress-induced inflammation.^43^ Sphingomyelinase induces phosphatidylserine externalization, providing a platform for ADAM17 substrate access.^44^ This multilayered regulation enables ADAM17 to function as a context-sensitive integrator of diverse pathological signals.

### Other ADAM/ADAMTS members as integrative hubs

Beyond ADAM17, other family members also integrate multiple pathological axes, although the underlying signaling mechanisms remain less clear. ADAMTS4 connects inflammation with ECM remodeling. The proinflammatory cytokines TNF-α and IL-1β synergistically induce ADAMTS4 expression in CMs and FBs. ADAMTS4 cleaves ECM proteoglycan versican, generating bioactive fragments that worsen cardiac dysfunction.^52^ It also directly cleaves EDA-fibronectin and LTBP-1, releasing latent TGF-β from the ECM. Activated TGF-β promotes FB-to-myoFB transition, collagen deposition, and diastolic dysfunction. Treatment with the ADAMTS4 inhibitor PPS reduces ADAMTS4 mRNA expression and versicanase activity, improving left ventricular systolic function.^47^ ADAMTS13 integrates thrombosis, inflammation, and fibrosis through VWF cleavage. As a specific VWF-cleaving protease, ADAMTS13 exerts cardioprotective effects through multiple interconnected mechanisms. By cleaving VWF multimers, it reduces VWF-mediated leukocyte infiltration and subsequent NETosis, limiting infarct size following I/R.^49^ In PO-induced HF, ADAMTS13 deficiency elevates VWF, activating neutrophils via the SLC44A2 receptor and promoting NET formation. NETs damage CMs through the TLR4-p38 MAPK-PGC-1α pathway, impairing mitochondrial function.^22^ ADAMTS13 treatment disrupts this VWF-SLC44A2-NET axis. Simultaneously, by reducing VWF-mediated platelet recruitment, ADAMTS13 decreases active TGF-β1 levels and cardiac fibrosis.^54^ Thus, ADAMTS13 modulates inflammation, thrombosis, NETosis, and fibrosis through VWF cleavage, preserving left ventricular function and reducing remodeling.

## ADAMTS family regulation of angiogenesis in HF

**Figure 8:**
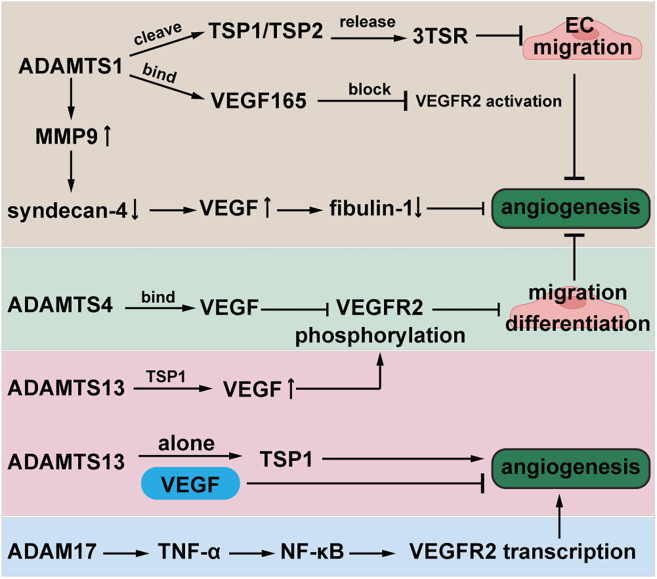
ADAM/ADAMTS family regulation of angiogenesis in HF ADAMTS1 inhibits angiogenesis through TSP1/2 cleavage (releasing antiangiogenic 3TSR fragments) and direct VEGF165 binding, suppressing VEGFR2 activation. ADAMTS4 similarly blocks VEGF-induced VEGFR2 phosphorylation and EC migration. Conversely, ADAMTS13 promotes angiogenesis via its TSP1 repeats in the absence of exogenous VEGF but sequesters VEGF and inhibits angiogenesis when exogenous VEGF is present. ADAM17 promotes post-MI angiogenesis by shedding TNF-α, activating NF-κB-mediated VEGFR2 transcription. 3TSR: three thrombospondin-1 type 1 repeats.

Research on angiogenesis during HF has primarily focused on the ADAMTS family, crucial for vascular remodeling due to their actions on basement membrane substrates.^76^ Their roles in angiogenesis involve interactions with VEGF, as shown in Figure 8. ADAMTS1 inhibits angiogenesis through two distinct mechanisms. Firstly, it proteolytically cleaves TSP1 and TSP2, releasing antiangiogenic fragments such as thrombospondin type-1 repeat 3 (3TSR), suppressing EC migration.^77^^,^^78^ Secondly, it directly binds to the carboxyl-terminal domain of VEGF165, blocking VEGFR2 activation.^79^^,^^80^ Additionally, ADAMTS1 exhibits functional synergy with syndecan-4 in regulating EC adhesion, migration, and angiogenesis. Knockdown of either ADAMTS1 or syndecan-4 enhances cellular responsiveness to VEGFA164, promoting microvessel outgrowth and EC migration in aortic ring assays. This increased migration depends on ECM changes, particularly reduced fibulin-1 expression, indicating coordinated regulation involving ADAMTS1, syndecan-4, MMP9, and fibulin-1.^81^ Similarly, EC-expressed ADAMTS4 binds VEGF, inhibiting VEGF-induced VEGFR2 phosphorylation, differentiation, and migration in human dermal microvascular ECs (HuDMECs), identifying it as another antiangiogenic factor.^82^ Conversely, CM-derived ADAM17 promotes post-MI angiogenesis by shedding TNF-α, activating NF-κB-mediated VEGFR2 transcription.^75^ ADAMTS13 has dual angiogenic roles via its TSP1 domain; alone, it promotes angiogenesis but inhibits VEGF-induced angiogenesis.^83^ Further research revealed that ADAMTS13 rapidly upregulates VEGF expression and enhances VEGFR2 phosphorylation in ECs, with TSP1 repeats 2–8 identified as the essential structural motif mediating these effects.^84^

## Roles of the ADAM and ADAMTS families in other CVDs

ADAM and ADAMTS proteases contribute to multiple CVDs, including AS, coronary artery disease (CAD), thrombosis, valvular diseases, and vascular wall pathologies, which ultimately lead to HF, highlighting their importance as both mechanistic mediators and therapeutic targets. ADAM17 promotes myocardial dysfunction by cleaving DSG2, thereby disrupting intercalated disc integrity; this effect is reversible with acute ADAM17 inhibition.^85^ ADAMTS6 mutations result in embryonic lethality with double-outlet right ventricle and septal defects.^86^^,^^87^ ADAMTS9 deficiency disrupts cardiovascular morphogenesis via versican degradation.^88^ ADAMTS19 mutations drive progressive valvular disease.^27^^,^^89^ ADAMTSL6 dysregulation promotes aortic aneurysms by disrupting ECM homeostasis.^30^ ADAMTS7 acts as a positive regulator of EC angiogenesis by degrading TSP, thereby promoting EC migration and tube formation.^90^ It also promotes AS and modulates plaque vulnerability through the dysregulation of the TIMP-1/MMP-9 axis.^91^^,^^92^ ADAMTSL2 upregulation in microvascular ECs induces metabolic reprogramming and fibrosis.^93^ Genetic variants at the ADAMTSL4 locus are associated with an increased risk of spontaneous coronary artery dissection.^94^ However, some ADAMTS family members exert cardioprotective effects. ADAMTS18 deficiency exacerbates AS in hyperlipidaemic models.^95^ Downregulation of ADAMTS18 in the atrial endocardium during atrial fibrillation promotes thrombus formation.^96^ ADAMTS13 deficiency promotes endothelial VWF accumulation and plaque progression in AS.^97^ Reduced ADAMTS13 activity leads to a VWF/ADAMTS13 imbalance, increasing thrombosis risk in congenital heart disease.^98^

## ADAM and ADAMTS as diagnostic and prognostic biomarkers in CVDs

Beyond their pathophysiological roles, dysregulated expression of specific ADAM/ADAMTS members serves as a clinically valuable biomarker for precursor CVDs, providing critical insights into early diagnosis, risk stratification, and outcome prediction. In CAD, elevated plasma ADAMTS5 levels are positively correlated with disease severity and independently predict major adverse cardiovascular events. This finding is based on a single-center retrospective study of 157 patients and supports its potential as a diagnostic and prognostic biomarker, pending further validation.^99^ In another single-center study of 154 patients with AMI, elevated plasma ADAMTS7 independently predicted adverse left ventricular remodeling, establishing it as a specific biomarker for this process.^100^ Based on an individual patient data meta-analysis of five case-control studies, including 1501 MI cases and 2258 controls, low plasma ADAMTS13 levels are independently associated with increased MI risk. No dose-response relationship was observed, and the association was stronger in females than in males.^101^ For carotid AS, genome-wide association studies identify ADAMTS9 as significantly associated with carotid intima-media thickness across multiple phenotypes. This suggests its utility in detecting subclinical AS, with a meta-analysis of over 100,000 individuals confirming this association.^102^ ADAM8 is a direct HF biomarker. Elevated plasma levels correlate with HFrEF and HFmrEF and predict impaired cardiac function and poor outcomes. This finding was validated in a single-center prospective cohort of 298 patients.^103^ This growing body of evidence demonstrates the progression of ADAMTS biomarkers from discovery in single-center cohorts to validation in larger populations and, in some cases, integration into large-scale meta-analyses. However, most candidates remain at the validation stage, and standardized assay protocols and prospective studies in diverse populations are required before clinical implementation.

## Regulation of ADAM/ADAMTS activity and specificity

### Direct inhibition

Endogenous inhibitors provide a physiological framework for selective protease control. As an endogenous inhibitor, TIMP3 selectively inhibits ADAM and ADAMTS, family members. TIMP3 inhibits ADAM10, 12, 17, 28, and 33. The N-terminal domain alone effectively inhibits ADAM12 and 17, with ADAM17 dimers exhibiting higher affinity for TIMP3. Full-length TIMP3 is required for effective inhibition of ADAM10. The non-catalytic domains of ADAMs modulate this inhibitory effect. For ADAMTS1, 2, 4, and 5, TIMP3 primarily mediates inhibition by binding its N-terminal domain to their catalytic centers. The C-terminal domains of ADAMTS4 and ADAMTS5 strengthen their binding to TIMP3, and ECM glycosaminoglycans further enhance TIMP3’s inhibitory activity against ADAMTS5.^104^ TIMP2 inhibits ADAM12 in a subtype-specific and structure-dependent manner through selective interaction between TIMP2’s N-terminal domain and ADAM12’s catalytic domain. The C-terminal non-catalytic domains of ADAM12 (Dis, Cys-rich, and EGF-like) negatively regulate this interaction.^105^^,^^106^^,^^107^ Alpha2-macroglobulin (α_2_M) serves as an endogenous inhibitor of ADAMTS4 and ADAMTS5 through a distinct trapping mechanism. ADAMTS4 and ADAMTS5 cleave the bait region at Met^690^-Gly^691^, triggering a conformational change that physically encapsulates the protease in an irreversible 1:1 complex.^108^^,^^109^ α_2_M also acts as both a substrate and inhibitor for ADAMTS7 and ADAMTS12, as it can be cleaved by these two enzymes.^109^^,^^110^

Exogenous inhibitors exploit domain-specific features to achieve greater selectivity compared to traditional MMP inhibitors.^8^^,^^104^^,^^111^^,^^112^ Small-molecule inhibitors can be classified by their mechanism of action. Zinc-binding compounds include GI 254023×, which demonstrates over 100-fold selectivity for ADAM10 by matching its S1′ pocket, and GW280264×, which inhibits ADAM10 and ADAM17 with similar potency.^111^^,^^113^^,^^114^ Arylsulfonamide 3a chelates ADAMTS7’s active-site zinc via its hydroxamate group, while its *p*-trifluoromethyl biphenyl moiety fits into the S1′ pocket, achieving 12-fold selectivity over ADAMTS5.^115^ BAY-9835, the first orally bioavailable ADAMTS7 inhibitor, employs a hydantoin zinc-binding group and achieves an IC_50_ of 6 nM with more than 1000-fold selectivity over ADAMTS4, ADAMTS5, and multiple MMPs.^116^^,^^117^ The non-zinc-binding inhibitor compound 4b selectively targets the disintegrin domain of ADAMTS5 through hydrogen bonding with K532 and K533, exhibiting no inhibitory activity against ADAMTS4.^118^ Although inhibitors targeting the hypervariable-loop exosites of ADAMTS4/5 spacer domains are currently unavailable, this approach could resolve selectivity limitations of catalytic-site inhibitors.^119^ Monoclonal antibodies inherently offer selectivity via protein-protein interactions. GLPG1972 binds ADAMTS5’s catalytic domain with a dissociation constant of 0.08 nM and has entered Phase I clinical trials for osteoarthritis.^120^ GSK2394002 allosterically inhibits ADAMTS5 by simultaneously interacting with its catalytic and disintegrin-like domains, stabilizing the enzyme in an inactive conformation.^112^

### Transcriptional and epigenetic regulation

Non-coding RNAs modulate ADAM/ADAMTS expression by targeting specific mRNA regions. Quercetin and fisetin in Yiqi Yangxue Formula upregulate endogenous LncRNA-UFC1, which acts as a sponge for miR-34a, resulting in reduced ADAMTS5 expression.^121^ miR-29a directly binds the 3′ untranslated region of ADAM12 mRNA, recruiting it to the Argonaut-2 silencing complex and inhibiting translation. Elevated miR-29a suppresses ADAM12-mediated angiogenesis in peripheral artery disease. The miR-29a antagomir competitively displaces ADAM12 mRNA in a dose-dependent manner without affecting ADAM17, confirming binding specificity.^122^ After intra-articular injection, exogenous siRNAs targeting MMP13 and ADAMTS5 achieve precise gene silencing through post-transcriptional regulation, mediated by complementary mRNA binding and activation of the RNA interference pathway.^123^

### Post-translational modifications (PTMs)

Oxidative PTMs have been primarily investigated in MMP-2 and MMP-9, where cysteine-switch oxidation or autocatalytic activation regulates proteolytic activity in cardiovascular injury.^8^ In contrast, direct evidence linking oxidative modifications to ADAM or ADAMTS family members remains limited. In CVDs, ROS induced by I/R directly activate MMP-2 through junctophilin-2 degradation.^8^^,^^124^ Phosphorylation has been clearly demonstrated for ADAM17. Polo-like kinase 2 (PLK2) binds the Ser^794^-Ala^810^ segment in ADAM17’s cytoplasmic tail via its Polo box domains and directly phosphorylates Ser^794^. This modification induces conformational changes that activate sheddase activity, promoting ectodomain shedding of pro-TNF and TNF receptors. Lipopolysaccharide stimulation upregulates PLK2, enhancing ADAM17-mediated inflammatory factor release. The PLK2 inhibitor BI 2536 blocks this pathway and reduces inflammation.^125^ Peptide-based modulation offers another regulatory mechanism. The C-terminal cleavage product of VWF-73 (Met^1606^-Arg^1668^) acts as an allosteric inhibitor of ADAMTS13 by binding its spacer domain.^126^^,^^127^ Aging-induced upregulation of miR-34a-5p suppresses membrane-associated RING-CH 8 (MARCHF8), reducing ubiquitination and degradation of ADAM10. Accumulation of ADAM10 promotes EC senescence and dysfunction, contributing to AS progression.^128^ Unlike MMPs, inhibitors targeting PTMs of ADAM/ADAMTS family members have been rarely explored.

## Opportunities and challenges in targeting ADAM/ADAMTS proteases in CVDs

ADAM/ADAMTS proteases exhibit subtype-specific pathological roles and defined regulatory networks that contribute to key pathophysiological processes in CVDs, including AS, HF, MI, ventricular remodeling, and inflammation.^8^^,^^15^^,^^35^^,^^60^^,^^100^^,^^126^

### Limitations of broad-spectrum MMP inhibitors

Clinical setbacks with first-generation broad-spectrum MMP inhibitors highlight significant concerns for targeting ADAM/ADAMTS, demonstrating limitations of non-selective inhibition.^129^^,^^130^ Initially, the dose-limiting toxicity of early MMP inhibitors arose from their zinc-chelating mechanism, which non-selectively targeted conserved catalytic domains, affecting both pathological and physiological MMPs. Physiological MMPs are essential for morphogenesis, tissue remodeling, bone metabolism, and wound healing.^131^^,^^132^^,^^133^ Systemic administration caused dose-limiting musculoskeletal syndrome (MSS). Marimastat treatment in small-cell lung cancer did not improve survival and negatively impacted patients’ quality of life due to MSS. Despite promising preclinical efficacy in tumor and myocardial remodeling models, marimastat failed in phase III trials because of MSS.^134^^,^^135^ Another limitation was inadequate assessment of the therapeutic index (effective-to-toxic dose ratio). Since MMP inhibitors are cytostatic rather than cytotoxic, clinical doses were deliberately lowered to prevent MSS, resulting in plasma concentrations later in discussion therapeutic levels.^129^^,^^130^ Despite promising preclinical results where broad-spectrum MMP inhibition (PD166793) reduced LV dilation, wall stress, and stiffness post-MI in a porcine model,^136^ a subsequent clinical trial using the selective oral MMP inhibitor PG-116800 showed no improvement in LV remodeling or clinical outcomes for patients with ST-segment elevation MI.^137^^,^^138^ These findings highlight two critical principles for translational success: (1) subtype-specific targeting to avoid disrupting physiological proteolytic balance; and (2) a strategic shift toward targeting subtype-exclusive binding sites. These insights may inform contemporary ADAM/ADAMTS inhibitor design and translational strategies.

### Advantages of ADAM/ADAMTS as therapeutic targets for CVDs

Unlike broad-spectrum MMPs, ADAM/ADAMTS proteases offer enhanced functional specificity. Key subtypes perform non-redundant, context-specific roles in CVDs, thereby lowering toxicity risks and supporting translational development. ADAMTS7 modulates atherosclerotic plaque formation by degrading TIMP-1^92^; ADAM17 mediates inflammatory myocardial injury via TNF-α release^35^; ADAMTS13 maintains thrombotic balance through VWF cleavage^23^; and ADAMTS5 deficiency leads to versican accumulation and cardiac dysfunction.^59^ This specificity removes the need for broad inhibition, thus avoiding unintended disruption of physiological processes. Furthermore, ADAM/ADAMTS activity is regulated by well-defined endogenous mechanisms, including TIMP-3, α_2_M, and epigenetic pathways such as lncRNA-UFC1/miR-34a and miRNA-29a.^104^^,^^108^^,^^121^ Engineered N-terminal TIMP-3 mutants selectively inhibit ADAM17 and ADAMTS4/5 without affecting MMPs, offering a natural template for selective inhibitor design.^139^^,^^140^ These regulatory networks allow ADAM/ADAMTS inhibitors to target pathological processes specifically, reducing the risk of MMP-like toxicity.

### Future translational strategies and challenges

Novel inhibitors now move beyond traditional zinc chelation, achieving high subtype selectivity through mechanisms such as domain-specific targeting, allosteric inhibition, and epigenetic regulation. Peptide-based inhibitors can target poorly conserved loops to achieve domain specificity.^127^ Biologics targeting ancillary-domain exosites provide alternative subtype-specific inhibition strategies.^109^^,^^118^ Epigenetic mechanisms, including promoter hypermethylation of ADAMTS1, 5, 8, and 12, represent endogenous regulatory layers with therapeutic potential.^109^ Besides subtype selectivity and reduced systemic toxicity, timing emerges as a critical factor influencing therapeutic efficacy. In MI, short-term ADAM17 inhibition post-injury reduces infarct expansion and improves cardiac function; however, pre-injury inhibition is ineffective or even detrimental, indicating a narrow therapeutic window.^141^ In vascular remodeling, ADAMTS7 expression peaks 7 days post-injury, defining an optimal intervention window for inhibiting neointima formation.^142^^,^^143^ For immune thrombotic thrombocytopenic purpura linked to ADAMTS13 deficiency, early administration (≤3 days after diagnosis) of the nanobody caplacizumab reduces plasma exchange requirements without delaying ADAMTS13 recovery; later treatment initiation loses this benefit.^144^ These findings underscore that intervention timing is as crucial as target selection. The Yiqi Yangxue formula modulates the lncRNA-UFC1/miR-34a/ADAMTS5 axis, exemplifying multi-target strategies.^121^ Future research should prioritize phenotype-guided targeting, exploit epigenetic and endogenous regulatory networks, and develop targeted delivery systems. Biomarker-driven patient selection based on these mechanisms may overcome therapeutic index limitations encountered by earlier MMP inhibitors.

Despite significant progress, several challenges hinder the clinical translation of ADAM/ADAMTS-targeted therapies. First, structural conservation of catalytic domains among MMPs, ADAMs, and ADAMTS predisposes small-molecule inhibitors to off-target effects. Although biologics offer better selectivity, their parenteral administration is less convenient. Peptide inhibitors combine selectivity with synthetic ease but have short half-lives and poor membrane permeability.^127^ Second, an incomplete understanding of target biology complicates therapeutic design. Certain ADAMTS members have multiple substrates, making single-target interventions inadequate. Functional redundancy among family members may cause compensatory upregulation during chronic inhibition, as observed with ADAM9 and ADAM15 following sustained ADAM17 blockade.^85^ Third, targeted delivery systems must overcome immunogenicity and distinguish pathological from healthy tissues. Optimal therapeutic windows remain poorly defined; ADAM17 inhibition benefits acute injury but not chronic dosing.^85^^,^^141^ The field also lacks standardized cardiovascular endpoints and validated biomarkers for patient stratification. Addressing these challenges will require improved inhibitors, deeper insight into disease-specific protease biology, and precise determination of intervention timing.

## Conclusion

ADAM and ADAMTS proteases serve as key regulators in HF pathogenesis. They integrate inflammatory, fibrotic, metabolic, and hypertrophic processes through horizontal and vertical integration. Their dual functionality, involving both detrimental and protective effects, highlights a critical balance in cardiac adaptation to stress, beyond conventional neurohormonal mechanisms. Functional heterogeneity within this protease family prevents generalizations from single members to the entire group. Each protease requires evaluation within specific disease contexts, pathological stages, and substrate repertoires. These insights offer novel therapeutic opportunities, particularly for patients with treatment-resistant HF, by targeting specific protease hubs instead of broad pathways. Future research should prioritize three complementary strategies. Clarifying the spatiotemporal dynamics of proteases in human cardiac tissue will define context-specific functions throughout disease progression. Developing subtype-selective inhibitors and synergistic combination therapies will translate mechanistic insights into precision treatments. Integrating multi-omics approaches will facilitate phenotype-specific targeting and biomarker-guided patient stratification. Collectively, these efforts provide a framework for precision HF management through targeted interventions focused on the ADAM/ADAMTS regulatory network.

## Acknowledgments

This work was supported by the 10.13039/501100019971Luzhou Science and Technology Bureau [grant nos. 2024JYJ009 and 2024JYJ051] and Innovation and Entrepreneurship Project for College Students of 10.13039/501100014895Southwest Medical University [202410632015 and S202510632246].

## Author contributions

X.W. and Y.H.: wrote the initial manuscript draft. Y.W., Y.L., X.D., Y.X., and L.D.: organized the literature. C.L.: modified the format. D.Z.: designed and conceptualized the study, drew the diagrams, and revised the manuscript. All authors approved the eventual version for submission. D.Z. and X.W.: acquired the funding.

## Declaration of interests

The authors declare no competing interests.

## Declaration of generative AI and AI-assisted technologies in the writing process

During the preparation of this work the authors used Grammarly and DeepSeek in order to improve readability and language. After using this tool/service, the authors reviewed and edited the content as needed and take full responsibility for the content of the published article.

## References

[1] Groenewegen A., Rutten F.H., Mosterd A., Hoes A.W. (2020). Epidemiology of heart failure. Eur. J. Heart Fail..

[2] Khan M.S., Shahid I., Bennis A., Rakisheva A., Metra M., Butler J. (2024). Global epidemiology of heart failure. Nat. Rev. Cardiol..

[3] Feng J., Zhang Y., Zhang J. (2024). Epidemiology and Burden of Heart Failure in Asia. JACC. Asia.

[4] Chen-Izu Y., Banyasz T., Shaw J.A., Izu L.T. (2025). The Heart Is a Smart Pump: Mechanotransduction Mechanisms of the Frank-Starling Law and the Anrep Effect. Annu. Rev. Physiol..

[5] Zhang D., Wen Q., Zhang R., Kou K., Lin M., Zhang S., Yang J., Shi H., Yang Y., Tan X. (2024). From Cell to Gene: Deciphering the Mechanism of Heart Failure With Single-Cell Sequencing. Adv. Sci..

[6] Zhang C., Teng X., Cao Q., Deng Y., Yang M., Wang L., Rui D., Ling X., Wei C., Chen Y. (2025). Gut microbiota dysbiosis exacerbates heart failure by the LPS-TLR4/NF-κB signalling axis: mechanistic insights and therapeutic potential of TLR4 inhibition. J. Transl. Med..

[7] Abudouwayiti A., Li Y.X., Aimaier S., Zheng Y.-Y., Mahemuti A. (2025). Segatella exacerbates chronic heart failure via TLR4/NF-κB pathway and therapeutic potential of low-carbohydrate diet. Cell Death Discov..

[8] Tanase D.M., Valasciuc E., Anton I.-B., Gosav E.M., Dima N., Cucu A.I., Costea C.F., Floria D.E., Hurjui L.L., Tarniceriu C.C. (2025). Matrix Metalloproteinases: Pathophysiologic Implications and Potential Therapeutic Targets in Cardiovascular Disease. Biomolecules.

[9] Lin Y.-J., Kwok C.-F., Juan C.-C., Hsu Y.-P., Shih K.-C., Chen C.-C., Ho L.-T. (2014). Angiotensin II enhances endothelin-1-induced vasoconstriction through upregulating endothelin type A receptor. Biochem. Biophys. Res. Commun..

[10] Rodrigues K.E., Pontes M.H.B., Cantão M.B.S., Prado A.F. (2024). The role of matrix metalloproteinase-9 in cardiac remodeling and dysfunction and as a possible blood biomarker in heart failure. Pharmacol. Res..

[11] Radosinska J., Barancik M., Vrbjar N. (2017). Heart failure and role of circulating MMP-2 and MMP-9. Panminerva Med..

[12] Seegar T.C., Blacklow S.C. (2019). Domain integration of ADAM family proteins: Emerging themes from structural studies. Exp. Biol. Med..

[13] Kilic T., Okuno K., Eguchi S., Kassiri Z. (2022). Disintegrin and Metalloproteinases (ADAMs [A Disintegrin and Metalloproteinase] and ADAMTSs [ADAMs With a Thrombospondin Motif]) in Aortic Aneurysm. Hypertension.

[14] Wang Z., Li W., Chen S., Tang X.X. (2023). Role of ADAM and ADAMTS proteases in pathological tissue remodeling. Cell Death Discov..

[15] Chen Q., Li Y., Bie B., Zhao B., Zhang Y., Fang S., Li S., Zhang Y. (2023). P38 MAPK activated ADAM17 mediates ACE2 shedding and promotes cardiac remodeling and heart failure after myocardial infarction. Cell Commun. Signal..

[16] Horiuchi K., Zhou H.M., Kelly K., Manova K., Blobel C.P. (2005). Evaluation of the contributions of ADAMs 9, 12, 15, 17, and 19 to heart development and ectodomain shedding of neuregulins beta1 and beta2. Dev. Biol..

[17] Farber G., Parks M.M., Lustgarten Guahmich N., Zhang Y., Monette S., Blanchard S.C., Di Lorenzo A., Blobel C.P. (2019). ADAM10 controls the differentiation of the coronary arterial endothelium. Angiogenesis.

[18] Xu T., Jiang S., Liu T., Han S., Wang Y. (2024). ADAM10 Alleviates Hypoxia/Reoxygenation-Induced Cardiomyocyte Injury by Activating the Notch Signaling Pathway. Cell Biochem. Biophys..

[19] Andersson H.M., Siegerink B., Luken B.M., Crawley J.T.B., Algra A., Lane D.A., Rosendaal F.R. (2012). High VWF, low ADAMTS13, and oral contraceptives increase the risk of ischemic stroke and myocardial infarction in young women. Blood.

[20] Ozawa K., Packwood W., Muller M.A., Qi Y., Xie A., Varlamov O., McCarty O.J., Chung D., López J.A., Lindner J.R. (2024). Removal of endothelial surface-associated von villebrand factor suppresses accelerate datherosclerosis after myocardial infarction. J. Transl. Med..

[21] Dhanesha N., Prakash P., Doddapattar P., Khanna I., Pollpeter M.J., Nayak M.K., Staber J.M., Chauhan A.K. (2016). Endothelial Cell–Derived von Willebrand Factor Is the Major Determinant That Mediates von Willebrand Factor–Dependent Acute Ischemic Stroke by Promoting Postischemic Thrombo-Inflammation. Arterioscler. Thromb. Vasc. Biol..

[22] Mang G., Chen J., Sun P., Ma R., Du J., Wang X., Cui J., Yang M., Tong Z., Yan X. (2024). Von Willebrand factor exacerbates heart failure through formation of neutrophil extracellular traps. Eur. Heart J..

[23] Newnham M., South K., Bleda M., Auger W.R., Barberà J.A., Bogaard H., Bunclark K., Cannon J.E., Delcroix M., Hadinnapola C. (2019). The ADAMTS13–VWF axis is dysregulated in chronic thromboembolic pulmonary hypertension. Eur. Respir. J..

[24] Rypdal K.B., Apte S.S., Lunde I.G. (2024). Emerging roles for the ADAMTS-like family of matricellular proteins in cardiovascular disease through regulation of the extracellular microenvironment. Mol. Biol. Rep..

[25] Lambert J., Edwards D.R., Apte S.S. (2020). ADAMTS Proteases: Methods and Protocols.

[26] Sheng S., Zhang S. (2025). The Dual Role of ADAMTS1 in Cardiovascular Remodeling: Balancing Extracellular Matrix Homeostasis and Pathological States. J. Cardiovasc. Dev. Dis..

[27] Santamaria S., de Groot R. (2020). ADAMTS proteases in cardiovascular physiology and disease. Open Biol..

[28] Zhang P., Shen M., Fernandez-Patron C., Kassiri Z. (2016). ADAMs family and relatives in cardiovascular physiology and pathology. J. Mol. Cell. Cardiol..

[29] Dubail J., Apte S.S. (2015). Insights on ADAMTS proteases and ADAMTS-like proteins from mammalian genetics. Matrix Biol..

[30] Mougin Z., Huguet Herrero J., Boileau C., Le Goff C. (2021). ADAMTS Proteins and Vascular Remodeling in Aortic Aneurysms. Biomolecules.

[31] Zhong S., Khalil R.A. (2019). A Disintegrin and Metalloproteinase (ADAM) and ADAM with thrombospondin motifs (ADAMTS) family in vascular biology and disease. Biochem. Pharmacol..

[32] Yang H., Khalil R.A. (2022). ADAM and ADAMTS disintegrin and metalloproteinases as major factors and molecular targets in vascular malfunction and disease. Adv. Pharmacol..

[33] Bacchetti R., Yuan S., Rainero E. (2024). ADAMTS Proteases: Their Multifaceted Role in the Regulation of Cancer Metastasis. Dis. Res..

[34] Kelwick R., Desanlis I., Wheeler G.N., Edwards D.R. (2015). The ADAMTS (A Disintegrin and Metalloproteinase with Thrombospondin motifs) family. Genome Biol..

[35] Adu-Amankwaah J., Adzika G.K., Adekunle A.O., Ndzie Noah M.L., Mprah R., Bushi A., Akhter N., Huang F., Xu Y., Adzraku S.Y. (2021). ADAM17, A Key Player of Cardiac Inflammation and Fibrosis in Heart Failure Development During Chronic Catecholamine Stress. Front. Cell Dev. Biol..

[36] Yu Y., Cao Y., Bell B., Chen X., Weiss R.M., Felder R.B., Wei S.G. (2019). Brain TACE (Tumor Necrosis Factor-α-Converting Enzyme) Contributes to Sympathetic Excitation in Heart Failure Rats. Hypertension.

[37] Yu Y., Xue B., Irfan N.M., Beltz T., Weiss R.M., Johnson A.K., Felder R.B., Wei S.G. (2022). Reducing brain TACE activity improves neuroinflammation and cardiac function in heart failure rats. Front. Physiol..

[38] Ji Z., Guo J., Wang M., Zhang R., Dong R., Sheng Z., Zuo P., Zhu K., Li Y., Yao Y. (2025). ADAM8 in macrophages exacerbates sepsis-induced cardiomyopathy by impeding efferocytosis. Front. Immunol..

[39] Ji Z., Guo J., Zhang R., Zuo W., Xu Y., Qu Y., Tao Z., Li X., Li Y., Yao Y., Ma G. (2025). ADAM8 deficiency in macrophages promotes cardiac repair after myocardial infarction via ANXA2-mTOR-autophagy pathway. J. Adv. Res..

[40] Cabrera-Fuentes H.A., Ruiz-Meana M., Barreto G., Serebruany V.L., Sánchez-Vega J.T., Pérez-Campos E., Kostin S., Böning A., Jarquín González E.E., Al-Suhaimi E.A. (2025). Extracellular RNA drives TNF-α/TNF-receptor-1 mediated cardiac ischemia/reperfusion injury: Mechanistic insights and therapeutic potential of RNase1. Pharmacol. Res..

[41] Patel V.B., Zhong J.-C., Grant M.B., Oudit G.Y. (2016). Role of the ACE2/Angiotensin 1–7 Axis of the Renin–Angiotensin System in Heart Failure. Circ. Res..

[42] Xie L., Xue F., Cheng C., Sui W., Zhang J., Meng L., Lu Y., Xiong W., Bu P., Xu F. (2024). Cardiomyocyte-specific knockout of ADAM17 alleviates doxorubicin-induced cardiomyopathy via inhibiting TNFα–TRAF3–TAK1–MAPK axis. Signal Transduct. Targeted Ther..

[43] Adu-Amankwaah J., Bushi A., Tan R., Adekunle A.O., Adzika G.K., Ndzie Noah M.L., Nadeem I., Adzraku S.Y., Koda S., Mprah R. (2023). Estradiol mitigates stress-induced cardiac injury and inflammation by downregulating ADAM17 via the GPER-1/PI3K signaling pathway. Cell. Mol. Life Sci..

[44] Sommer A., Düppe M., Baumecker L., Kordowski F., Büch J., Chico J.F., Fritsch J., Schütze S., Adam D., Sperrhacke M. (2017). Extracellular sphingomyelinase activity impairs TNF-α-induced endothelial cell death via ADAM17 activation and TNF receptor 1 shedding. Oncotarget.

[45] van der Vorst E.P.C., Maas S.L., Theodorou K., Peters L.J.F., Jin H., Rademakers T., Gijbels M.J., Rousch M., Jansen Y., Weber C. (2023). Endothelial ADAM10 controls cellular response to oxLDL and its deficiency exacerbates atherosclerosis with intraplaque hemorrhage and neovascularization in mice. Front. Cardiovasc. Med..

[46] Klapproth E., Witt A., Klose P., Wiedemann J., Vavilthota N., Künzel S.R., Kämmerer S., Günscht M., Sprott D., Lesche M. (2022). Targeting cardiomyocyte ADAM10 ectodomain shedding promotes survival early after myocardial infarction. Nat. Commun..

[47] Vistnes M., Aronsen J.M., Lunde I.G., Sjaastad I., Carlson C.R., Christensen G. (2014). Pentosan polysulfate decreases myocardial expression of the extracellular matrix enzyme ADAMTS4 and improves cardiac function in vivo in rats subjected to pressure overload by aortic banding. PLoS One.

[48] Hanby H.A., Zheng X.L. (2013). Biochemistry and physiological functions of ADAMTS7 metalloprotease. Adv. Biochem..

[49] Savchenko A.S., Borissoff J.I., Martinod K., De Meyer S.F., Gallant M., Erpenbeck L., Brill A., Wang Y., Wagner D.D. (2014). VWF-mediated leukocyte recruitment with chromatin decondensation by PAD4 increases myocardial ischemia/reperfusion injury in mice. Blood.

[50] Frangogiannis N.G. (2024). TGF-β as a therapeutic target in the infarcted and failing heart: cellular mechanisms, challenges, and opportunities. Expert Opin. Ther. Targets.

[51] Yao L., Shao W., Chen Y., Wang S., Huang D. (2022). Suppression of ADAM8 attenuates angiotensin II-induced cardiac fibrosis and endothelial-mesenchymal transition via inhibiting TGF-β1/Smad2/Smad3 pathways. Exp. Anim..

[52] Vistnes M., Erusappan P.M., Sasi A., Nordén E.S., Bergo K.K., Romaine A., Lunde I.G., Zhang L., Olsen M.B., Øgaard J. (2023). Inhibition of the extracellular enzyme A disintegrin and metalloprotease with thrombospondin motif 4 prevents cardiac fibrosis and dysfunction. Cardiovasc. Res..

[53] Yao Y., Hu C., Song Q., Li Y., Da X., Yu Y., Li H., Clark I.M., Chen Q., Wang Q.K. (2020). ADAMTS16 activates latent TGF-β, accentuating fibrosis and dysfunction of the pressure-overloaded heart. Cardiovasc. Res..

[54] Witsch T., Martinod K., Sorvillo N., Portier I., De Meyer S.F., Wagner D.D. (2018). Recombinant Human ADAMTS13 Treatment Improves Myocardial Remodeling and Functionality After Pressure Overload Injury in Mice. J. Am. Heart Assoc..

[55] Rypdal K.B., Erusappan P.M., Melleby A.O., Seifert D.E., Palmero S., Strand M.E., Tønnessen T., Dahl C.P., Almaas V., Hubmacher D. (2021). The extracellular matrix glycoprotein ADAMTSL2 is increased in heart failure and inhibits TGFβ signalling in cardiac fibroblasts. Sci. Rep..

[56] Rypdal K.B., Olav Melleby A., Robinson E.L., Li J., Palmero S., Seifert D.E., Martin D., Clark C., López B., Andreassen K. (2022). ADAMTSL3 knock-out mice develop cardiac dysfunction and dilatation with increased TGFβ signalling after pressure overload. Commun. Biol..

[57] Nakamura Y., Kita S., Tanaka Y., Fukuda S., Obata Y., Okita T., Kawachi Y., Tsugawa-Shimizu Y., Fujishima Y., Nishizawa H. (2020). A disintegrin and metalloproteinase 12 prevents heart failure by regulating cardiac hypertrophy and fibrosis. Am. J. Physiol. Heart Circ. Physiol..

[58] Hoeft K., Koch L., Ziegler S., Zhang L., Luetke S., Tanzer M.C., Mohanta D., Schumacher D., Schreibing F., Long Q. (2024). ADAMTS12 promotes fibrosis by restructuring extracellular matrix to enable activation of injury-responsive fibroblasts. J. Clin. Investig..

[59] Barallobre-Barreiro J., Radovits T., Fava M., Mayr U., Lin W.-Y., Ermolaeva E., Martínez-López D., Lindberg E.L., Duregotti E., Daróczi L. (2021). Extracellular Matrix in Heart Failure: Role of ADAMTS5 in Proteoglycan Remodeling. Circulation.

[60] Mead T.J., Bhutada S., Peruzzi N., Adegboye J., Seifert D.E., Cahill E., Drinko J., Donnellan E., Guggiliam A., Popovic Z. (2025). ADAMTS7, a target in atherosclerosis, cooperates with its homolog ADAMTS12 to protect against myxomatous valve degeneration. J. Mol. Cell. Cardiol..

[61] Chute M., Aujla P.K., Li Y., Jana S., Zhabyeyev P., Rasmuson J., Owen C.A., Abraham T., Oudit G.Y., Kassiri Z. (2022). ADAM15 is required for optimal collagen cross-linking and scar formation following myocardial infarction. Matrix Biol..

[62] Omura J., Satoh K., Kikuchi N., Satoh T., Kurosawa R., Nogi M., Ohtsuki T., Al-Mamun M.E., Siddique M.A.H., Yaoita N. (2019). ADAMTS8 Promotes the Development of Pulmonary Arterial Hypertension and Right Ventricular Failure: A Possible Novel Therapeutic Target. Circ. Res..

[63] Zha Y., Li Y., Ge Z., Wang J., Jiao Y., Zhang J., Zhang S. (2022). ADAMTS8 Promotes Cardiac Fibrosis Partly Through Activating EGFR Dependent Pathway. Front. Cardiovasc. Med..

[64] Schlüter K.-D., Steinert I., Preissner K., Bencsik P., Sárközy M., Csonka C., Ferdinandy P., Schulz R., Schlüter K.D., Schreckenberg R., Weber P. (2017). Mechanism and consequences of the shift in cardiac arginine metabolism following ischaemia and reperfusion in rats. Thromb. Haemost..

[65] Wang W., He Y., Liang D., Zhou L., Yang T., Gu L., Du C., Wang S., Wang H., Wang L., Wang Q. (2025). Fibroblast-secreted ADAMTSL2 promotes cardiac repair after myocardial infarction by activating LRP6/β-catenin signaling. Cell. Signal..

[66] Fedak P.W.M., Moravec C.S., McCarthy P.M., Altamentova S.M., Wong A.P., Skrtic M., Verma S., Weisel R.D., Li R.K. (2006). Altered expression of disintegrin metalloproteinases and their inhibitor in human dilated cardiomyopathy. Circulation.

[67] Aujla P.K., Hu M., Hartley B., Kranrod J.W., Viveiros A., Kilic T., Owen C.A., Oudit G.Y., Seubert J.M., Julien O., Kassiri Z. (2023). Loss of ADAM15 Exacerbates Transition to Decompensated Myocardial Hypertrophy and Dilation Through Activation of the Calcineurin Pathway. Hypertension.

[68] Asakura M., Kitakaze M., Takashima S., Liao Y., Ishikura F., Yoshinaka T., Ohmoto H., Node K., Yoshino K., Ishiguro H. (2002). Cardiac hypertrophy is inhibited by antagonism of ADAM12 processing of HB-EGF: metalloproteinase inhibitors as a new therapy. Nat. Med..

[69] Fan D., Takawale A., Shen M., Samokhvalov V., Basu R., Patel V., Wang X., Fernandez-Patron C., Seubert J.M., Oudit G.Y., Kassiri Z. (2016). A Disintegrin and Metalloprotease-17 Regulates Pressure Overload-Induced Myocardial Hypertrophy and Dysfunction Through Proteolytic Processing of Integrin β1. Hypertension.

[70] Ren L., Wu C., Yang K., Chen S., Ye P., Wu J., Zhang A., Huang X., Wang K., Deng P. (2018). A Disintegrin and Metalloprotease-22 Attenuates Hypertrophic Remodeling in Mice Through Inhibition of the Protein Kinase B Signaling Pathway. J. Am. Heart Assoc..

[71] Xiang M., Luo H., Wu J., Ren L., Ding X., Wu C., Chen J., Chen S., Zhang H., Yu L. (2018). ADAM23 in Cardiomyocyte Inhibits Cardiac Hypertrophy by Targeting FAK - AKT Signaling. J. Am. Heart Assoc..

[72] Wang X., Chen W., Zhang J., Khan A., Li L., Huang F., Qiu Z., Wang L., Chen X. (2017). Critical Role of ADAMTS2 (A Disintegrin and Metalloproteinase With Thrombospondin Motifs 2) in Cardiac Hypertrophy Induced by Pressure Overload. Hypertension.

[73] Rau C.D., Romay M.C., Tuteryan M., Wang J.J.C., Santolini M., Ren S., Karma A., Weiss J.N., Wang Y., Lusis A.J. (2017). Systems Genetics Approach Identifies Gene Pathways and Adamts2 as Drivers of Isoproterenol-Induced Cardiac Hypertrophy and Cardiomyopathy in Mice. Cell Syst..

[74] Wang W., Wu C., Ren L., Bao Y., Han Y., Li C., Li Y. (2020). MiR-30e-5p is sponged by Kcnq1ot1 and represses Angiotensin II-induced hypertrophic phenotypes in cardiomyocytes by targeting ADAM9. Exp. Cell Res..

[75] Fan D., Takawale A., Shen M., Wang W., Wang X., Basu R., Oudit G.Y., Kassiri Z. (2015). Cardiomyocyte A Disintegrin And Metalloproteinase 17 (ADAM17) Is Essential in Post-Myocardial Infarction Repair by Regulating Angiogenesis. Circ. Heart Fail..

[76] Rodríguez-Manzaneque J.C., Fernández-Rodríguez R., Rodríguez-Baena F.J., Iruela-Arispe M.L. (2015). ADAMTS proteases in vascular biology. Matrix Biol..

[77] Lee N.V., Sato M., Annis D.S., Loo J.A., Wu L., Mosher D.F., Iruela-Arispe M.L. (2006). ADAMTS1 mediates the release of antiangiogenic polypeptides from TSP1 and 2. EMBO J..

[78] Zhang K., Li M., Yin L., Fu G., Liu Z. (2020). Role of thrombospondin-1 and thrombospondin-2 in cardiovascular diseases. Int. J. Mol. Med..

[79] Luque A., Carpizo D.R., Iruela-Arispe M.L. (2003). ADAMTS1/METH1 inhibits endothelial cell proliferation by direct binding and sequestration of VEGF165. J. Biol. Chem..

[80] Lungu C.N., Mehedinti M.C. (2023). Molecular Motifs in Vascular Morphogenesis: Vascular Endothelial Growth Factor A (VEGFA) as the Leading Promoter of Angiogenesis. Int. J. Mol. Sci..

[81] Lambert J., Makin K., Akbareian S., Johnson R., Alghamdi A.A.A., Robinson S.D., Edwards D.R. (2020). ADAMTS-1 and syndecan-4 intersect in the regulation of cell migration and angiogenesis. J. Cell Sci..

[82] Hsu Y.P., Staton C.A., Cross N., Buttle D.J. (2012). Anti-angiogenic properties of ADAMTS-4 in vitro. Int. J. Exp. Pathol..

[83] Lee M., Rodansky E.S., Smith J.K., Rodgers G.M. (2012). ADAMTS13 promotes angiogenesis and modulates VEGF-induced angiogenesis. Microvasc. Res..

[84] Lee M., Keener J., Xiao J., Long Zheng X., Rodgers G.M. (2015). ADAMTS13 and its variants promote angiogenesis via upregulation of VEGF and VEGFR2. Cell. Mol. Life Sci..

[85] Shoykhet M., Waschke J., Yeruva S. (2023). Cardiomyocyte cohesion is increased after ADAM17 inhibition. Front. Cell Dev. Biol..

[86] Li Y., Klena N.T., Gabriel G.C., Liu X., Kim A.J., Lemke K., Chen Y., Chatterjee B., Devine W., Damerla R.R. (2015). Global genetic analysis in mice unveils central role for cilia in congenital heart disease. Nature.

[87] Prins B.P., Mead T.J., Brody J.A., Sveinbjornsson G., Ntalla I., Bihlmeyer N.A., van den Berg M., Bork-Jensen J., Cappellani S., Van Duijvenboden S. (2018). Exome-chip meta-analysis identifies novel loci associated with cardiac conduction, including ADAMTS6. Genome Biol..

[88] Kern C.B., Wessels A., McGarity J., Dixon L.J., Alston E., Argraves W.S., Geeting D., Nelson C.M., Menick D.R., Apte S.S. (2010). Reduced versican cleavage due to Adamts9 haploinsufficiency is associated with cardiac and aortic anomalies. Matrix Biol..

[89] Wünnemann F., Ta-Shma A., Preuss C., Leclerc S., van Vliet P.P., Oneglia A., Thibeault M., Nordquist E., Lincoln J., Scharfenberg F. (2020). Loss of ADAMTS19 causes progressive non-syndromic heart valve disease. Nat. Genet..

[90] Pu X., Chan K., Yang W., Xiao Q., Zhang L., Moore A.D., Liu C., Webb T.R., Caulfield M.J., Samani N.J. (2020). Effect of a coronary-heart-disease-associated variant of ADAMTS7 on endothelial cell angiogenesis. Atherosclerosis.

[91] Bauer R.C., Tohyama J., Cui J., Cheng L., Yang J., Zhang X., Ou K., Paschos G.K., Zheng X.L., Parmacek M.S. (2015). Knockout of Adamts7, a novel coronary artery disease locus in humans, reduces atherosclerosis in mice. Circulation.

[92] Sharifi M.A., Wierer M., Dang T.A., Milic J., Moggio A., Sachs N., von Scheidt M., Hinterdobler J., Müller P., Werner J. (2023). ADAMTS-7 Modulates Atherosclerotic Plaque Formation by Degradation of TIMP-1. Circ. Res..

[93] Trenson S., Hermans H., Craps S., Pokreisz P., de Zeeuw P., Van Wauwe J., Gillijns H., Veltman D., Wei F., Caluwé E. (2021). Cardiac Microvascular Endothelial Cells in Pressure Overload-Induced Heart Disease. Circ. Heart Fail..

[94] Saw J., Yang M.L., Trinder M., Tcheandjieu C., Xu C., Starovoytov A., Birt I., Mathis M.R., Hunker K.L., Schmidt E.M. (2020). Chromosome 1q21.2 and additional loci influence risk of spontaneous coronary artery dissection and myocardial infarction. Nat. Commun..

[95] Zhu R., Cheng M., Lu T., Yang N., Ye S., Pan Y.H., Hong T., Dang S., Zhang W. (2018). A Disintegrin and Metalloproteinase with Thrombospondin Motifs 18 Deficiency Leads to Visceral Adiposity and Associated Metabolic Syndrome in Mice. Am. J. Pathol..

[96] Cerveró J., Segura V., Macías A., Gavira J.J., Montes R., Hermida J. (2012). Atrial fibrillation in pigs induces left atrial endocardial transcriptional remodelling. Thromb. Haemost..

[97] Ozawa K., Muller M.A., Varlamov O., Tavori H., Packwood W., Mueller P.A., Xie A., Ruggeri Z., Chung D., López J.A., Lindner J.R. (2020). Proteolysis of Von Willebrand Factor Influences Inflammatory Endothelial Activation and Vascular Compliance in Atherosclerosis. JACC, Basic Transl. Sci..

[98] Katneni U.K., Holcomb D.D., Hernandez N.E., Hamasaki-Katagiri N., Hunt R.C., Bar H., Ibla J.C., Kimchi-Sarfaty C. (2020). In silico features of ADAMTS13 contributing to plasmatic ADAMTS13 levels in neonates with congenital heart disease. Thromb. Res..

[99] Pan H., Liu J. (2023). Role of ADAMTS-5 Expression in the Prognosis of Patients with Coronary Artery Disease: A Single Retrospective Analysis. Int. J. Clin. Med..

[100] Wu W., Zhou Y., Li Y., Li J., Ke Y., Wang Y., Zheng J. (2015). Association between plasma ADAMTS-7 levels and ventricular remodeling in patients with acute myocardial infarction. Eur. J. Med. Res..

[101] Maino A., Siegerink B., Lotta L.A., Crawley J.T.B., le Cessie S., Leebeek F.W.G., Lane D.A., Lowe G.D.O., Peyvandi F., Rosendaal F.R. (2015). Plasma ADAMTS-13 levels and the risk of myocardial infarction: an individual patient data meta-analysis. J. Thromb. Haemostasis.

[102] Yeung M.W., Wang S., van de Vegte Y.J., Borisov O., van Setten J., Snieder H., Verweij N., Said M.A., van der Harst P. (2022). Twenty-Five Novel Loci for Carotid Intima-Media Thickness: A Genome-Wide Association Study in >45 000 Individuals and Meta-Analysis of >100 000 Individuals. Arterioscler. Thromb. Vasc. Biol..

[103] Ji Z., Guo J., Xu Y., Zuo W., Zhang R., Carvalho A., Zhang X., Tao Z., Li X., Yao Y., Ma G. (2024). Prognostic value of a disintegrin and metalloproteinase Domain-8 in heart failure. Heliyon.

[104] Fan D., Kassiri Z. (2020). Biology of Tissue Inhibitor of Metalloproteinase 3 (TIMP3), and Its Therapeutic Implications in Cardiovascular Pathology. Front. Physiol..

[105] Jacobsen J., Visse R., Sørensen H.P., Enghild J.J., Brew K., Wewer U.M., Nagase H. (2008). Catalytic properties of ADAM12 and its domain deletion mutants. Biochemistry.

[106] Kveiborg M., Jacobsen J., Lee M.-H., Nagase H., Wewer U.M., Murphy G. (2010). Selective inhibition of ADAM12 catalytic activity through engineering of tissue inhibitor of metalloproteinase 2 (TIMP-2). Biochem. J..

[107] Peeney D., Liu Y., Lazaroff C., Gurung S., Stetler-Stevenson W.G. (2022). Unravelling the distinct biological functions and potential therapeutic applications of TIMP2 in cancer. Carcinogenesis.

[108] Tortorella M.D., Arner E.C., Hills R., Easton A., Korte-Sarfaty J., Fok K., Wittwer A.J., Liu R.Q., Malfait A.M. (2004). Alpha2-macroglobulin is a novel substrate for ADAMTS-4 and ADAMTS-5 and represents an endogenous inhibitor of these enzymes. J. Biol. Chem..

[109] Rose K.W.J., Taye N., Karoulias S.Z., Hubmacher D. (2021). Regulation of ADAMTS Proteases. Front. Mol. Biosci..

[110] Luan Y., Kong L., Howell D.R., Ilalov K., Fajardo M., Bai X.H., Di Cesare P.E., Goldring M.B., Abramson S.B., Liu C.J. (2008). Inhibition of ADAMTS-7 and ADAMTS-12 degradation of cartilage oligomeric matrix protein by alpha-2-macroglobulin. Osteoarthr. Cartil..

[111] Malemud C.J. (2019). Inhibition of MMPs and ADAM/ADAMTS. Biochem. Pharmacol..

[112] Larkin J., Lohr T.A., Elefante L., Shearin J., Matico R., Su J.L., Xue Y., Liu F., Genell C., Miller R.E. (2015). Translational development of an ADAMTS-5 antibody for osteoarthritis disease modification. Osteoarthr. Cartil..

[113] Ludwig A., Hundhausen C., Lambert M.H., Broadway N., Andrews R.C., Bickett D.M., Leesnitzer M.A., Becherer J.D. (2005). Metalloproteinase inhibitors for the disintegrin-like metalloproteinases ADAM10 and ADAM17 that differentially block constitutive and phorbol ester-inducible shedding of cell surface molecules. Comb. Chem. High Throughput Screen..

[114] Minond D. (2020). Novel Approaches and Challenges of Discovery of Exosite Modulators of a Disintegrin and Metalloprotease 10. Front. Mol. Biosci..

[115] Cuffaro D., Burkhard T., Bernardoni B.L., Di Leo R., Zhang X., Galati S., Tuccinardi T., Macchia M., Rossello A., Santamaria S. (2024). Design, synthesis and biological evaluation of arylsulfonamides as ADAMTS7 inhibitors. RSC Med. Chem..

[116] Meibom D., Wasnaire P., Beyer K., Broehl A., Cancho-Grande Y., Elowe N., Henninger K., Johannes S., Jungmann N., Krainz T. (2024). BAY-9835: Discovery of the First Orally Bioavailable ADAMTS7 Inhibitor. J. Med. Chem..

[117] Khera A.V., Kathiresan S. (2017). Genetics of coronary artery disease: discovery, biology and clinical translation. Nat. Rev. Genet..

[118] Santamaria S., Cuffaro D., Nuti E., Ciccone L., Tuccinardi T., Liva F., D'Andrea F., de Groot R., Rossello A., Ahnström J. (2021). Exosite inhibition of ADAMTS-5 by a glycoconjugated arylsulfonamide. Sci. Rep..

[119] Santamaria S., Yamamoto K., Teraz-Orosz A., Koch C., Apte S.S., de Groot R., Lane D.A., Ahnström J. (2019). Exosites in Hypervariable Loops of ADAMTS Spacer Domains control Substrate Recognition and Proteolysis. Sci. Rep..

[120] Brebion F., Gosmini R., Deprez P., Varin M., Peixoto C., Alvey L., Jary H., Bienvenu N., Triballeau N., Blanque R. (2021). Discovery of GLPG1972/S201086, a Potent, Selective, and Orally Bioavailable ADAMTS-5 Inhibitor for the Treatment of Osteoarthritis. J. Med. Chem..

[121] Zhao T., Wang X., Li Z., Qin D. (2025). Yiqi Yangxue formula inhibits cartilage degeneration in knee osteoarthritis by regulating LncRNA-UFC1/miR-34a/MMP-13 axis. J. Ethnopharmacol..

[122] Lamin V., Verry J., Dokun O.S., Kronemberger A., Wong T., Lira V.A., Dokun A.O. (2022). microRNA-29a Regulates ADAM12 Through Direct Interaction With ADAM12 mRNA and Modulates Postischemic Perfusion Recovery. J. Am. Heart Assoc..

[123] Hoshi H., Akagi R., Yamaguchi S., Muramatsu Y., Akatsu Y., Yamamoto Y., Sasaki T., Takahashi K., Sasho T. (2017). Effect of inhibiting MMP13 and ADAMTS5 by intra-articular injection of small interfering RNA in a surgically induced osteoarthritis model of mice. Cell Tissue Res..

[124] Chan B.Y.H., Roczkowsky A., Cho W.J., Poirier M., Lee T.Y.T., Mahmud Z., Schulz R. (2019). Junctophilin-2 is a target of matrix metalloproteinase-2 in myocardial ischemia-reperfusion injury. Basic Res. Cardiol..

[125] Schwarz J., Schmidt S., Will O., Koudelka T., Köhler K., Boss M., Rabe B., Tholey A., Scheller J., Schmidt-Arras D. (2014). Polo-like Kinase 2, a Novel ADAM17 Signaling Component, Regulates Tumor Necrosis Factor α Ectodomain Shedding. J. Biol. Chem..

[126] Di Stasio E., Lancellotti S., Peyvandi F., Palla R., Mannucci P.M., De Cristofaro R. (2008). Mechanistic studies on ADAMTS13 catalysis. Biophys. J..

[127] Pluda S., Mazzocato Y., Angelini A. (2021). Peptide-Based Inhibitors of ADAM and ADAMTS Metalloproteinases. Front. Mol. Biosci..

[128] Qian Z., Huang Y., Yang N., Fang Z., Zhang Y., Huang Y., Luo M., Ji T., Chen Z., Gao S. (2025). miR-34a-5p/MARCHF8/ADAM10 axis in the regulation of vascular endothelial cell dysfunction and senescence. Mech. Ageing Dev..

[129] Fields G.B. (2019). The Rebirth of Matrix Metalloproteinase Inhibitors: Moving Beyond the Dogma. Cells.

[130] Raeeszadeh-Sarmazdeh M., Do L.D., Hritz B.G. (2020). Metalloproteinases and Their Inhibitors: Potential for the Development of New Therapeutics. Cells.

[131] Sreesada P., Nair B.G., Krishnan B., Krishnan B., Amrutha R., Chavan Y., Alfia H., Jyothis A., Venugopal P., Aradhya R., Suravajhala P. (2025). Matrix metalloproteinases: Master regulators of tissue morphogenesis. Gene.

[132] Paiva K.B.S., Granjeiro J.M., Khalil R.A. (2017). Progress in Molecular Biology and Translational Science.

[133] Ayuk S.M., Abrahamse H., Houreld N.N. (2016). The Role of Matrix Metalloproteinases in Diabetic Wound Healing in relation to Photobiomodulation. J. Diabetes Res..

[134] Shepherd F.A., Giaccone G., Seymour L., Debruyne C., Bezjak A., Hirsh V., Smylie M., Rubin S., Martins H., Lamont A. (2002). Prospective, randomized, double-blind, placebo-controlled trial of marimastat after response to first-line chemotherapy in patients with small-cell lung cancer: a trial of the National Cancer Institute of Canada-Clinical Trials Group and the European Organization for Research and Treatment of Cancer. J. Clin. Oncol..

[135] Winer A., Adams S., Mignatti P. (2018). Matrix Metalloproteinase Inhibitors in Cancer Therapy: Turning Past Failures Into Future Successes. Mol. Cancer Therapeut..

[136] Yarbrough W.M., Mukherjee R., Brinsa T.A., Dowdy K.B., Scott A.A., Escobar G.P., Joffs C., Lucas D.G., Crawford F.A., Spinale F.G. (2003). Matrix metalloproteinase inhibition modifies left ventricular remodeling after myocardial infarction in pigs. J. Thorac. Cardiovasc. Surg..

[137] Hudson M.P., Armstrong P.W., Ruzyllo W., Brum J., Cusmano L., Krzeski P., Lyon R., Quinones M., Theroux P., Sydlowski D. (2006). Effects of selective matrix metalloproteinase inhibitor (PG-116800) to prevent ventricular remodeling after myocardial infarction: results of the PREMIER (Prevention of Myocardial Infarction Early Remodeling) trial. J. Am. Coll. Cardiol..

[138] Li Y., Cheng K. (2025). Delaying Drug Release. JACC, Basic Transl. Sci..

[139] Wei S., Kashiwagi M., Kota S., Xie Z., Nagase H., Brew K. (2005). Reactive Site Mutations in Tissue Inhibitor of Metalloproteinase-3 Disrupt Inhibition of Matrix Metalloproteinases but Not Tumor Necrosis Factor-α-converting Enzyme. J. Biol. Chem..

[140] Lim N.H., Kashiwagi M., Visse R., Jones J., Enghild J.J., Brew K., Nagase H. (2010). Reactive-site mutants of N-TIMP-3 that selectively inhibit ADAMTS-4 and ADAMTS-5: biological and structural implications. Biochem. J..

[141] Li Y., Al Rimon R., Wang F., Li H., Epelman S., Tallquist M.D., Westover L., Oudit G.Y., Kassiri Z. (2026). Temporal inhibition of ADAM17 in fibroblasts reduces stiffness and promotes vascularization following myocardial infarction. Cardiovasc. Res..

[142] Wang L., Zheng J., Bai X., Liu B., Liu C.-J., Xu Q., Zhu Y., Wang N., Kong W., Wang X. (2009). ADAMTS-7 Mediates Vascular Smooth Muscle Cell Migration and Neointima Formation in Balloon-Injured Rat Arteries. Circ. Res..

[143] Gong Z., Huang J., Wang D., Yang S., Ma Z., Fu Y., Ma Q., Kong W. (2023). ADAMTS-7 deficiency attenuates thoracic aortic aneurysm and dissection in mice. J. Mol. Med..

[144] Mingot-Castellano M.E., García-Candel F., Martínez-Nieto J., García-Arroba J., de la Rubia-Comos J., Gómez-Seguí I., Paciello-Coronel M.L., Valcárcel-Ferreiras D., Jiménez M., Cid J. (2024). ADAMTS13 recovery in acute thrombotic thrombocytopenic purpura after caplacizumab therapy. Blood.

